# Supplementary material for: COVID-19 immune signatures in Uganda persist in HIV co-infection and diverge by pandemic phase
Source: Nat Commun. 2024 Feb 17;15:1475. doi: 10.1038/s41467-024-45204-3 (PMC10874401; doi:10.1038/s41467-024-45204-3)
Supplement: Supplementary file 1 — Supplemental Information [file 41467_2024_45204_MOESM1_ESM.pdf]

Supplemental Information

Members of the Collaboration for Clinical and Laboratory Characterization of COVID-19 in Uganda are as follows: **National Influenza Centre, Department of Arbovirology, Emerging and Re-emerging Infectious Diseases, Uganda Virus Research Institute, Entebbe, Uganda** (Barnabas Bakamutumaho, Nicholas Owor, John Kayiwa, Mercy Haumba, Joyce Namulondo, Timothy Byaruhanga, Julius J. Lutwama, Joweria Nakaseegu, Jocelyn Kiconco, Annet Nankya, Irene Ataliba, Samuel Wavamuno); **Viral Hemorrhagic Fever Program, Uganda Virus Research Institute, Entebbe, Uganda** (Stephen Balinandi, Luke Nyakaruhuka, Jimmy Baluku, Alex Tumusiime, Jackson Kyondo, Sophia Mulei, Kilama Kamugisha, Gloria Akurut, Diana Nahamya); **Immunisable Diseases Unit/Expanded Programme on Immunisation Laboratory, Uganda Virus Research Institute, Entebbe, Uganda** (Peter Eliku, Phiona Tushabe, Joshua Buule, Joseph Gaizi, Prossy Namuwulya, Arnold Mugaga, Mary Nyacho, Henry Bukenya, Josephine Bwogi); **European and Developing Countries Clinical Trials Partnership-Eastern Africa Consortium for Clinical Research, Uganda Virus Research Institute, Entebbe, Uganda** (Benjamin Watyaba, Bernard Kikaire); **Entebbe Regional Referral Hospital, Entebbe, Uganda** (Moses Muwanga, Christopher Nsereko, Emmanuel Rwamutwe, Irene Nayiga, Stephen Kyebambe, Roselyn Mutonyi, Josephine Achan, Lucy Wanyenze, Alice Ndazarwe, Joseph Shinyale, Ruth Nakanjako, Richard Natuhwera, Annet Nsangi); **Uganda Peoples' Defence Forces, Kampala, Uganda and Ministry of Health, Kampala, Uganda** (Henry Kyobe Bosa); **Ministry of Health, Kampala, Uganda** (Felix Ocom); **Makerere University School of Public Health, Kampala, Uganda** (Noah Kiwanuka, Stevens Kisaka, James Arinaitwe); **Division of Pulmonary, Allergy, and Critical Care Medicine, Department of Medicine, Vagelos College of Physicians and Surgeons; Center for Infection and Immunity, Department of Epidemiology, Mailman School of Public Health; Columbia University, New York, NY, USA** (Max R. O'Donnell, Matthew J. Cummings).

Supplemental Methods

Classification of COVID-19 severity (based on World Health Organization criteria applicable to resource-limited settings[17])

Severity Classification	Criteria
Asymptomatic SARS-CoV-2 infection	Absence of all the following symptoms: fever, night sweats, cough, headache, sore throat, runny nose/rhinorrhea, shortness of breath, *plus* no signs of respiratory distress (chest indrawing, nasal flaring, grunting respirations)
Mild COVID-19	≥1 of the above symptoms in the absence of shortness of breath and signs of respiratory distress, *plus* oxygen saturation >90%, respiratory rate <30 breaths/min, and no receipt of oxygen therapy.
Moderate COVID-19	≥1 of the above symptoms *plus* shortness of breath, *without* signs of respiratory distress and oxygen saturation >90%, respiratory rate <30 breaths/min, and no receipt of oxygen therapy.
Severe COVID-19	≥1 of the following: (1) oxygen saturation ≤90%, (2) respiratory rate ≥30 breaths/minute, (3) ≥1 sign of respiratory distress, (4) receipt of oxygen-therapy.

Supplemental Tables

Supplemental Table 1: Immune mediator concentrations stratified by COVID-19 severity.

Mediator[a]	All patients N=306	Asymptomatic N=66	Mild N=149	Moderate N=21	Severe N=70	p-value[b]
**sCD40L**	3.77 [3.38, 3.99]	3.71 [3.19, 3.93]	3.73 [3.41, 3.95]	3.93 [3.74, 4.02]	3.86 [3.37, 4.06]	0.105
**EGF**	2.28 [1.95, 2.57]	2.32 [2.01, 2.67]	2.20 [1.84, 2.52]	2.40 [2.06, 2.55]	2.35 [2.09, 2.61]	0.139
**Eotaxin**	1.47 [1.24, 1.62]	1.48 [1.34, 1.60]	1.49 [1.23, 1.63]	1.52 [1.32, 1.65]	1.41 [1.17, 1.63]	0.506
**FGF-2**	1.81 [1.51, 2.11]	1.84 [1.50, 2.14]	1.75 [1.49, 2.09]	2.03 [1.73, 2.47]	1.83 [1.60, 2.09]	0.121
**FLT-3L**	1.38 [1.11, 1.62]	1.37 [1.13, 1.59]	1.38 [1.09, 1.62]	1.51 [1.25, 1.73]	1.37 [1.15, 1.62]	0.648
**Fractalkine**	2.09 [1.95, 2.29]	2.10 [1.97, 2.26]	2.04 [1.91, 2.27]	2.35 [2.12, 2.75]	2.15 [1.99, 2.33]	<0.001
**G-CSF[c]**	-0.18 [-0.18, 1.30]	-0.18 [-0.18, 1.18]	-0.18 [-0.18, 1.26]	-0.18 [-0.18, 1.49]	0.86 [-0.18, 1.52]	0.096
**GM-CSF[c]**	-1.00 [-1.00, -1.00]	-1.00 [-1.00, -1.00]	-1.00 [-1.00, -1.00]	-1.00 [-1.00, -1.00]	-1.00 [-1.00, -1.00]	0.062
**GRO-α/CXCL1**	2.01 [1.83, 2.17]	2.02 [1.82, 2.15]	2.02 [1.81, 2.16]	2.01 [1.89, 2.14]	2.00 [1.87, 2.22]	0.865
**IFN-α2**	1.82 [1.61, 2.04]	1.80 [1.51, 1.99]	1.81 [1.60, 2.03]	1.89 [1.60, 2.55]	1.83 [1.71, 2.03]	0.352
**IFN-γ**	-0.08 [-1.40, 0.69]	-0.37 [-1.40, 0.67]	-0.14 [-1.40, 0.62]	0.29 [-1.40, 1.21]	0.02 [-1.30, 0.69]	0.209
**IL-1a**	1.41 [1.15, 1.77]	1.42 [1.17, 1.80]	1.41 [1.10, 1.73]	1.77 [1.24, 2.34]	1.36 [1.19, 1.66]	0.175
**IL-1β**	1.44 [1.09, 1.78]	1.41 [1.07, 1.74]	1.39 [1.05, 1.75]	1.81 [1.16, 2.24]	1.48 [1.15, 1.79]	0.185
**IL-1Ra**	0.86 [0.54, 1.31]	0.81 [0.50, 1.19]	0.72 [0.49, 1.12]	1.38 [0.76, 1.63]	1.14 [0.77, 1.63]	<0.001
**IL-2**	0.01 [-0.62, 0.53]	-0.03 [-0.86, 0.59]	-0.02 [-0.62, 0.53]	0.26 [-0.23, 1.26]	0.02 [-0.50, 0.39]	0.307
**IL-3[c]**	-2.00 [-2.00, -2.00]	-2.00 [-2.00, -2.00]	-2.00 [-2.00, -2.00]	-2.00 [-2.00, -2.00]	-2.00 [-2.00, -0.77]	0.009
**IL-4**	0.20 [-0.11, 0.46]	0.13 [-0.20, 0.39]	0.10 [-0.28, 0.39]	0.28 [0.08, 0.53]	0.40 [0.17, 0.61]	<0.001
**IL-5**	0.71 [0.28, 1.12]	0.99 [0.52, 1.46]	0.72 [0.29, 1.07]	0.78 [0.24, 1.10]	0.50 [0.15, 1.00]	0.002
**IL-6**	0.49 [0.10, 1.13]	0.42 [0.09, 0.97]	0.33 [0.03, 0.69]	0.79 [0.24, 1.22]	1.01 [0.55, 1.45]	<0.001
**IL-7**	0.84 [0.51, 1.08]	0.69 [0.36, 0.97]	0.73 [0.43, 0.97]	0.87 [0.60, 1.06]	1.24 [0.93, 1.59]	<0.001
**IL-8**	1.78 [1.26, 2.37]	1.96 [1.26, 2.55]	1.73 [1.21, 2.22]	2.00 [1.37, 2.53]	1.67 [1.29, 2.47]	0.356
**IL-9**	0.59 [-0.33, 0.94]	0.59 [-0.33, 0.95]	0.51 [-0.33, 0.87]	0.87 [0.51, 1.17]	0.69 [-0.10, 0.98]	0.017
**IL-10**	0.60 [0.02, 1.16]	0.39 [-0.25, 0.85]	0.52 [-0.17, 0.85]	0.64 [0.38, 0.95]	1.24 [0.71, 1.58]	<0.001
**IL-12p40**	1.64 [1.27, 1.91]	1.72 [1.34, 1.92]	1.66 [1.39, 1.90]	1.89 [1.27, 2.22]	1.38 [0.96, 1.84]	0.010
**IL-12p70**	0.42 [0.29, 0.62]	0.47 [0.17, 0.64]	0.40 [0.26, 0.60]	0.56 [0.35, 0.83]	0.42 [0.33, 0.66]	0.206
**IL-13**	1.86 [1.65, 2.18]	1.85 [1.63, 2.24]	1.81 [1.60, 2.10]	2.03 [1.81, 2.57]	1.98 [1.75, 2.18]	0.018
**IL-15**	1.34 [1.11, 1.63]	1.28 [1.06, 1.53]	1.25 [1.03, 1.44]	1.44 [1.26, 1.90]	1.66 [1.36, 1.84]	<0.001
**IL-17A**	0.97 [0.57, 1.41]	0.94 [0.56, 1.56]	0.97 [0.53, 1.34]	1.21 [0.71, 1.79]	0.86 [0.60, 1.36]	0.259
**IL-17E/IL-25**	2.55 [2.27, 2.77]	2.55 [2.16, 2.82]	2.48 [2.18, 2.72]	2.74 [2.48, 2.97]	2.62 [2.39, 2.77]	0.030
**IL-17F**	0.83 [-0.25, 1.49]	0.42 [-0.25, 1.62]	0.78 [-0.25, 1.40]	0.70 [-0.25, 1.54]	1.12 [0.54, 1.52]	0.044
**IL-18**	1.68 [1.41, 1.93]	1.48 [1.20, 1.84]	1.66 [1.40, 1.92]	1.73 [1.47, 1.94]	1.79 [1.59, 2.08]	0.001
**IL-22**	1.81 [1.35, 2.06]	1.82 [1.43, 1.99]	1.70 [0.84, 1.99]	1.86 [1.71, 2.28]	1.95 [1.66, 2.13]	0.001
**IL-27**	3.15 [2.89, 3.37]	3.05 [2.86, 3.34]	3.14 [2.88, 3.35]	3.22 [2.96, 3.41]	3.28 [2.94, 3.50]	0.061
**IP-10/CXCL10**	2.25 [1.96, 2.60]	2.00 [1.82, 2.25]	2.24 [1.97, 2.51]	2.30 [1.98, 2.54]	2.82 [2.30, 3.78]	<0.001
**MCP-1**	2.35 [2.19, 2.48]	2.34 [2.22, 2.47]	2.36 [2.20, 2.47]	2.31 [2.10, 2.55]	2.35 [2.17, 2.52]	0.955
**MCP-3**	1.24 [1.08, 1.55]	1.18 [1.05, 1.54]	1.18 [1.06, 1.38]	1.74 [1.25, 2.07]	1.36 [1.15, 1.71]	<0.001
**M-CSF**	2.14 [1.87, 2.42]	2.08 [1.86, 2.51]	2.08 [1.78, 2.32]	2.25 [1.99, 2.48]	2.34 [2.11, 2.60]	<0.001
**MDC**	3.03 [2.85, 3.20]	3.04 [2.93, 3.22]	3.11 [2.95, 3.24]	3.00 [2.86, 3.13]	2.81 [2.61, 2.96]	<0.001
**MIG/CXCL9**	3.47 [3.24, 3.67]	3.44 [3.22, 3.63]	3.42 [3.22, 3.57]	3.50 [3.14, 3.68]	3.67 [3.38, 3.89]	<0.001
**MIP-1α/CCL3**	1.67 [1.45, 2.22]	1.73 [1.51, 2.40]	1.58 [1.40, 2.22]	1.73 [1.65, 2.19]	1.63 [1.49, 1.92]	0.156
**MIP-1β/CCL4**	1.83 [1.64, 2.07]	1.89 [1.72, 2.14]	1.80 [1.63, 2.00]	2.04 [1.77, 2.31]	1.84 [1.62, 2.10]	0.052
**PDGF-AA**	3.53 [3.39, 3.69]	3.49 [3.36, 3.68]	3.50 [3.38, 3.66]	3.64 [3.46, 3.73]	3.62 [3.43, 3.76]	0.046
**PDGF-AB/BB**	4.47 [4.42, 4.52]	4.48 [4.44, 4.53]	4.48 [4.42, 4.52]	4.51 [4.44, 4.58]	4.45 [4.39, 4.51]	0.006
**RANTES/CCL5**	3.37 [3.23, 3.48]	3.30 [3.18, 3.46]	3.37 [3.26, 3.47]	3.33 [3.16, 3.51]	3.40 [3.28, 3.53]	0.088
**TGF-α**	1.07 [0.76, 1.47]	1.03 [0.64, 1.45]	0.90 [0.67, 1.21]	0.97 [0.90, 1.59]	1.48 [1.14, 1.71]	<0.001
**TNF**	1.81 [1.64, 2.03]	1.71 [1.59, 2.02]	1.77 [1.62, 1.94]	1.94 [1.69, 2.37]	1.94 [1.76, 2.17]	<0.001
**Lymphotoxin-α**	1.37 [1.14, 1.65]	1.29 [1.15, 1.50]	1.24 [1.05, 1.48]	1.54 [1.25, 1.90]	1.60 [1.32, 1.80]	<0.001
**VEGF-A**	2.57 [2.31, 2.82]	2.56 [2.19, 2.71]	2.50 [2.19, 2.74]	2.63 [2.40, 2.89]	2.83 [2.56, 3.03]	<0.001

Legend: [a]Mediator concentrations presented as log_{10} pg/ml with median and interquartile range; [b]Reflects results of Kruskal-Wallis H test; [c]Large proportion of values below the lower limit of quantification (see Supplemental Table 15).

Supplemental Table 2: Association between COVID-19 severity and immune mediator concentrations in proportional odds models adjusted for age, sex, HIV co-infection, and SARS-CoV-2 variant phase (N=306).

Mediator	β (95% CI)[a]	Nominal p-value	BH-adjusted p-value
sCD40L	0.279 (-0.068 to 0.627)	0.115	0.197
EGF	-0.014 (-0.448 to 0.420)	0.949	0.949
Eotaxin	0.122 (-0.569 to 0.814)	0.729	0.778
FGF-2	0.099 (-0.307 to 0.505)	0.633	0.723
FLT-3L	0.110 (-0.396 to 0.616)	0.671	0.749
Fractalkine	0.599 (-0.075 to 1.274)	0.082	0.164
G-CSF[b]	0.268 (0.010 to 0.526)	0.041	0.112
GM-CSF[b]	0.254 (-0.002 to 0.511)	0.052	0.131
GROα/CXCL1	0.285 (-0.510 to 1.080)	0.482	0.578
IFN-α2	0.317 (-0.114 to 0.747)	0.150	0.219
IFN-γ	0.149 (-0.052 to 0.350)	0.146	0.219
IL-1α	0.075 (-0.296 to 0.446)	0.691	0.754
IL-1β	0.139 (-0.232 to 0.510)	0.464	0.571
IL-1Ra	0.500 (0.133 to 0.867)	0.008	0.030
IL-2	0.056 (-0.169 to 0.280)	0.628	0.723
IL-3[b]	0.126 (-0.166 to 0.417)	0.398	0.503
IL-4	0.507 (0.176 to 0.838)	0.003	0.013
IL-5	-0.707 (-1.078 to -0.336)	<0.001	<0.001
IL-6	0.347 (0.027 to 0.668)	0.034	0.102
IL-7	1.136 (0.664 to 1.607)	<0.001	<0.001
IL-8	-0.199 (-0.513 to 0.115)	0.214	0.293
IL-9	0.317 (-0.022 to 0.656)	0.067	0.142
IL-10	0.528 (0.295 to 0.760)	<0.001	<0.001
IL-12p40	-0.187 (-0.510 to 0.137)	0.258	0.344
IL-12p70	0.216 (-0.227 to 0.658)	0.339	0.440
IL-13	0.369 (-0.096 to 0.833)	0.120	0.199
IL-15	0.773 (0.258 to 1.287)	0.003	0.013
IL-17A	0.040 (-0.256 to 0.335)	0.792	0.809
IL-17E/IL-25	0.282 (-0.104 to 0.668)	0.152	0.219
IL-17F	0.198 (-0.045 to 0.441)	0.110	0.197
IL-18	0.821 (0.348 to 1.294)	<0.001	<0.001
IL-22	0.296 (-0.109 to 0.701)	0.152	0.219
IL-27	0.628 (0.022 to 1.232)	0.042	0.112
IP-10/CXCL10	1.090 (0.709 to 1.463)	<0.001	<0.001
MCP-1	-0.136 (-1.055 to 0.782)	0.771	0.805
MCP-3	0.619 (0.086 to 1.152)	0.022	0.070
M-CSF	0.332 (-0.012 to 0.675)	0.058	0.133
MDC	-1.561 (-2.415 to -0.707)	<0.001	<0.001
MIG/CXCL9	0.644 (-0.017 to 1.304)	0.056	0.133
MIP-1α/CCL3	-0.303 (-0.659 to 0.053)	0.096	0.184
MIP-1β/CCL4	-0.823 (-1.497 to -0.149)	0.017	0.058
PDGF-AA	0.792 (-0.060 to 1.644)	0.068	0.142
PDGF-AB/BB	-1.563 (-3.715 to 0.589)	0.155	0.219
RANTES	0.752 (-0.175 to 1.679)	0.112	0.197

TGF-α	0.687 (0.222 to 1.152)	0.004	0.016
TNF	0.997 (0.342 to 1.652)	0.003	0.013
LT-α	0.874 (0.335 to 1.413)	0.001	0.006
VEGF-A	0.815 (0.327 to 1.303)	0.001	0.006

Legend: [a]β reflects mediator regression coefficient in a proportional odds model with World Health Organization COVID-19 severity (asymptomatic, mild, moderate, severe) as ordinal dependent variable and log_{10} mediator concentration (continuous), age (continuous), sex (binary), HIV status (binary; 4 patients not known to be living with HIV but with missing rapid diagnostic tests analyzed as HIV negative), and SARS-CoV-2 variant phase (categorical) as independent variables; p-values reflect two-sided Wald test of mediator regression coefficient, adjusted using Benjamini-Hochberg method; [b]Large proportion of values below the lower limit of quantification (see Supplemental Table 15).

Abbreviations: BH: Benjamini-Hochberg, CI: confidence interval

Supplemental Table 3: Association between COVID-19 severity and immune mediator concentrations in proportional odds models adjusted for age, sex, HIV co-infection, SARS-CoV-2 variant phase, and pre-enrollment illness duration (N=240; 66 individuals with asymptomatic SARS-CoV-2 infection excluded).

Mediator	β (95% CI)[a]	Nominal p-value	BH-adjusted p-value
sCD40L	0.199 (-0.261 to 0.659)	0.396	0.475
EGF	0.504 (-0.161 to 1.168)	0.137	0.235
Eotaxin	0.412 (-0.501 to 1.325)	0.377	0.464
FGF-2	0.310 (-0.285 to 0.906)	0.307	0.421
FLT-3L	0.260 (-0.430 to 0.951)	0.460	0.539
Fractalkine	1.159 (0.233 to 2.086)	0.014	0.045
G-CSF[b]	0.340 (-0.013 to 0.694)	0.059	0.127
GM-CSF[b]	0.182 (-0.157 to 0.520)	0.292	0.412
GROα/CXCL1	-0.070 (-1.163 to 1.023)	0.900	0.900
IFN-α2	0.396 (-0.208 to 1.000)	0.199	0.303
IFN-γ	0.183 (-0.098 to 0.465)	0.202	0.303
IL-1α	0.125 (-0.340 to 0.647)	0.640	0.674
IL-1β	0.138 (-0.378 to 0.655)	0.600	0.670
IL-1Ra	0.682 (0.185 to 1.180)	0.007	0.031
IL-2	0.047 (-0.256 to 0.350)	0.760	0.776
IL-3[b]	0.492 (0.083 to 0.901)	0.018	0.054
IL-4	1.022 (0.430 to 1.614)	<0.001	<0.001
IL-5	-0.483 (-1.047 to 0.081)	0.093	0.186
IL-6	0.502 (0.082 to 0.922)	0.019	0.054
IL-7	1.526 (0.764 to 2.288)	<0.001	<0.001
IL-8	-0.109 (-0.534 to 0.315)	0.614	0.670
IL-9	0.385 (-0.091 to 0.862)	0.113	0.209
IL-10	0.713 (0.326 to 1.101)	<0.001	<0.001
IL-12p40	-0.442 (-0.880 to -0.005)	0.048	0.110
IL-12p70	0.321 (-0.312 to 0.954)	0.320	0.427
IL-13	0.733 (0.047 to 1.419)	0.036	0.086
IL-15	1.287 (0.531 to 2.044)	<0.001	<0.001
IL-17A	0.278 (-0.159 to 0.716)	0.212	0.308
IL-17E/IL-25	0.431 (-0.109 to 0.971)	0.118	0.210
IL-17F	0.334 (-0.016 to 0.684)	0.061	0.127
IL-18	0.501 (-0.228 to 1.229)	0.178	0.285
IL-22	0.832 (0.235 to 1.428)	0.006	0.029
IL-27	0.384 (-0.416 to 1.184)	0.347	0.438
IP-10/CXCL10	0.802 (0.359 to 1.244)	<0.001	<0.001
MCP-1	-0.332 (-1.499 to 0.834)	0.576	0.658
MCP-3	0.787 (0.104 to 1.470)	0.024	0.064
M-CSF	0.702 (0.152 to 1.252)	0.012	0.041
MDC	-2.383 (-3.591 to -1.176)	<0.001	<0.001
MIG/CXCL9	1.206 (0.319 to 2.093)	0.008	0.032
MIP-1α/CCL3	-0.367 (-0.867 to 0.132)	0.150	0.248
MIP-1β/CCL4	-0.432 (-1.308 to 0.443)	0.333	0.432
PDGF-AA	1.394 (0.168 to 2.620)	0.026	0.066

PDGF-AB/BB	-2.689 (-5.955 to 0.578)	0.107	0.205
RANTES	0.302 (-0.987 to 1.592)	0.646	0.674
TGF-α	1.440 (0.737 to 2.144)	<0.001	<0.001
TNF	1.155 (0.270 to 2.041)	0.011	0.041
LT-α	1.411 (0.618 to 2.204)	<0.001	<0.001
VEGF-A	1.420 (0.528 to 2.311)	0.002	0.011

Legend: [a]β reflects mediator regression coefficient in a proportional odds model with World Health Organization COVID-19 severity (mild, moderate, severe) as ordinal dependent variable and log_{10} mediator concentration (continuous), age (continuous), sex (binary), HIV status (binary; 4 patients not known to be living with HIV but with missing rapid diagnostic tests analyzed as HIV negative), SARS-CoV-2 variant phase (categorical) and illness duration at enrollment (continuous) as independent variables; p-values reflect two-sided Wald test of mediator regression coefficient, adjusted using Benjamini-Hochberg method; [b]Large proportion of values below the lower limit of quantification (see Supplemental Table 15).

Abbreviations: BH: Benjamini-Hochberg, CI: confidence interval

Supplemental Table 4: Immune mediator concentrations stratified by COVID-19 severity in persons living with HIV.

Mediator[a]	All PLWH N=33	Asymptomatic N=9	Mild N=14	Moderate N=6	Severe N=4	p-value[b]
**sCD40L**	3.61 [3.27, 3.76]	3.37 [3.15, 3.74]	3.57 [3.30, 3.66]	3.72 [3.23, 3.93]	3.85 [3.69, 4.02]	0.276
**EGF**	2.11 [1.60, 2.35]	1.87 [1.51, 2.19]	2.09 [1.83, 2.27]	2.31 [1.73, 2.52]	2.41 [2.29, 2.54]	0.194
**Eotaxin**	1.45 [1.17, 1.69]	1.48 [1.39, 1.60]	1.45 [1.13, 1.69]	1.40 [1.06, 1.56]	1.61 [1.25, 2.05]	0.784
**FGF-2**	1.64 [1.17, 2.03]	1.24 [1.13, 1.73]	1.48 [1.20, 2.16]	2.07 [1.69, 2.38]	1.78 [1.54, 2.00]	0.336
**FLT-3L**	1.32 [0.99, 1.59]	1.29 [0.74, 1.52]	1.20 [1.01, 1.60]	1.52 [1.30, 1.63]	1.45 [1.23, 1.67]	0.758
**Fractalkine**	2.03 [1.91, 2.24]	1.97 [1.80, 2.06]	2.02 [1.90, 2.08]	2.12 [2.03, 2.22]	2.43 [2.29, 2.52]	0.176
**G-CSF[c]**	-0.18 [-0.18, 1.11]	-0.18 [-0.18, -0.18]	-0.18 [-0.18, 1.12]	-0.18 [-0.18, -0.18]	1.72 [1.51, 1.99]	0.002
**GM-CSF[c]**	-1.00 [-1.00, -1.00]	-1.00 [-1.00, -1.00]	-1.00 [-1.00, -1.00]	-1.00 [-1.00, -1.00]	0.48 [-0.29, 1.27]	0.001
**GRO-α/CXCL1**	1.91 [1.68, 2.03]	1.78 [1.59, 1.86]	1.92 [1.73, 2.02]	1.90 [1.66, 1.98]	2.15 [2.04, 2.23]	0.097
**IFN-α2**	1.74 [1.41, 1.88]	1.32 [1.11, 1.83]	1.73 [1.59, 1.83]	1.66 [1.36, 1.88]	1.85 [1.77, 1.98]	0.255
**IFN-γ**	-0.57 [-1.40, 0.57]	-1.40 [-1.40, -0.39]	-0.55 [-1.40, 0.02]	-1.40 [-1.40, -0.31]	0.97 [0.73, 1.09]	0.066
**IL-1a**	1.31 [1.03, 1.64]	1.15 [0.98, 1.47]	1.21 [1.03, 1.62]	1.56 [1.27, 1.76]	1.42 [1.29, 1.56]	0.348
**IL-1β**	1.15 [0.82, 1.57]	0.90 [0.49, 1.17]	1.28 [0.77, 1.71]	1.25 [1.05, 1.77]	1.20 [1.17, 1.31]	0.427
**IL-1Ra**	0.67 [0.46, 0.98]	0.49 [0.03, 0.68]	0.64 [0.47, 0.80]	0.98 [0.53, 1.57]	1.45 [1.28, 1.70]	0.012
**IL-2**	-0.51 [-1.40, 0.20]	-0.89 [-1.70, -0.62]	-0.34 [-1.43, 0.33]	-0.05 [-0.42, 0.22]	-0.07 [-0.58, 0.17]	0.317
**IL-3[c]**	-2.00 [-2.00, -2.00]	-2.00 [-2.00, -2.00]	-2.00 [-2.00, -2.00]	-2.00 [-2.00, -2.00]	-2.00 [-2.00, -2.00]	0.212
**IL-4**	0.06 [-0.22, 0.35]	-0.26 [-1.70, 0.03]	0.04 [-0.19, 0.40]	0.23 [0.07, 0.39]	0.37 [0.32, 0.45]	0.046
**IL-5**	1.06 [0.50, 1.21]	1.24 [1.20, 1.48]	1.06 [0.70, 1.17]	0.37 [0.23, 0.68]	0.46 [0.27, 0.77]	0.029
**IL-6**	0.43 [0.07, 1.10]	0.32 [-0.15, 0.54]	0.20 [0.03, 0.42]	1.01 [0.81, 1.35]	1.65 [1.50, 1.77]	0.001
**IL-7**	0.82 [0.54, 1.23]	0.59 [0.32, 0.82]	0.75 [0.45, 1.04]	1.01 [0.81, 1.16]	1.54 [1.44, 1.66]	0.022
**IL-8**	1.54 [1.10, 2.29]	1.33 [0.98, 2.53]	1.54 [0.94, 1.73]	2.80 [1.82, 3.14]	1.43 [1.37, 1.89]	0.200
**IL-9**	0.30 [-0.33, 0.75]	-0.33 [-0.33, -0.33]	0.44 [-0.24, 0.90]	0.35 [0.23, 0.76]	0.22 [-0.12, 0.55]	0.287
**IL-10**	0.39 [-0.15, 1.17]	-0.15 [-0.37, 0.10]	0.43 [0.26, 0.91]	0.61 [-1.38, 1.30]	1.84 [1.64, 2.14]	0.005
**IL-12p40**	1.52 [1.09, 1.66]	1.43 [1.17, 1.54]	1.59 [1.16, 1.74]	1.34 [0.90, 1.80]	1.15 [1.08, 1.36]	0.812
**IL-12p70**	0.35 [0.20, 0.52]	0.22 [0.16, 0.38]	0.32 [0.23, 0.68]	0.43 [0.24, 0.55]	0.43 [0.38, 0.65]	0.195
**IL-13**	1.75 [1.47, 2.10]	1.75 [1.36, 2.10]	1.69 [1.54, 1.86]	1.67 [1.42, 1.79]	2.21 [2.16, 2.39]	0.146
**IL-15**	1.20 [1.04, 1.42]	1.13 [1.05, 1.18]	1.09 [1.00, 1.30]	1.29 [1.21, 1.40]	1.82 [1.60, 1.96]	0.023
**IL-17A**	0.76 [0.30, 1.14]	0.30 [0.14, 1.04]	0.74 [0.29, 1.12]	0.91 [0.64, 1.58]	0.78 [0.40, 1.15]	0.547
**IL-17E/IL-25**	2.30 [2.07, 2.78]	2.09 [1.84, 2.26]	2.32 [2.17, 2.43]	2.85 [1.46, 2.98]	2.81 [2.64, 2.86]	0.267
**IL-17F**	-0.25 [-0.25, 1.27]	-0.25 [-0.25, -0.25]	-0.25 [-0.25, 1.15]	0.46 [-0.25, 1.28]	1.10 [0.52, 1.44]	0.514
**IL-18**	1.69 [1.37, 1.87]	1.44 [1.37, 1.91]	1.68 [1.17, 1.86]	1.77 [1.59, 1.81]	1.74 [1.53, 1.97]	0.751
**IL-22**	1.69 [0.84, 2.07]	1.15 [0.84, 1.84]	1.65 [0.97, 1.75]	1.71 [1.37, 1.74]	2.17 [2.14, 2.27]	0.052
**IL-27**	3.08 [2.71, 3.23]	3.08 [2.71, 3.22]	3.09 [2.67, 3.28]	3.03 [2.67, 3.12]	3.79 [3.47, 3.88]	0.220
**IP-10/CXCL10**	2.47 [2.01, 2.59]	2.47 [2.04, 2.52]	2.45 [1.86, 2.61]	2.12 [2.02, 2.39]	4.20 [3.72, 4.43]	0.013
**MCP-1**	2.44 [2.28, 2.54]	2.42 [2.27, 2.45]	2.44 [2.34, 2.45]	2.33 [2.08, 2.59]	2.69 [2.54, 2.89]	0.084
**MCP-3**	1.09 [0.96, 1.70]	1.02 [0.93, 1.36]	1.06 [0.96, 1.16]	1.81 [1.10, 2.60]	1.70 [1.67, 1.80]	0.099
**M-CSF**	2.07 [1.86, 2.32]	2.09 [1.98, 2.16]	1.89 [1.77, 2.07]	2.04 [1.94, 2.36]	2.67 [2.53, 2.74]	0.044
**MDC**	2.97 [2.77, 3.17]	2.95 [2.71, 3.04]	3.14 [2.89, 3.25]	2.98 [2.94, 3.13]	2.77 [2.66, 2.88]	0.146
**MIG/CXCL9**	3.53 [3.25, 3.73]	3.43 [3.13, 3.63]	3.51 [3.35, 3.73]	3.65 [3.26, 3.68]	4.31 [3.89, 4.55]	0.167
**MIP-1α/CCL3**	1.49 [1.37, 1.68]	1.26 [1.20, 1.46]	1.45 [1.37, 1.59]	1.68 [1.66, 1.82]	1.50 [1.47, 1.92]	0.042
**MIP-1β/CCL4**	1.73 [1.62, 1.96]	1.78 [1.59, 1.96]	1.72 [1.66, 1.80]	2.12 [1.75, 2.42]	1.62 [1.59, 1.92]	0.441
**PDGF-AA**	3.42 [3.31, 3.54]	3.32 [3.27, 3.48]	3.45 [3.34, 3.54]	3.49 [3.42, 3.62]	3.59 [3.26, 3.88]	0.373
**PDGF-AB/BB**	4.43 [4.37, 4.49]	4.47 [4.34, 4.49]	4.44 [4.41, 4.48]	4.43 [4.43, 4.54]	4.33 [4.31, 4.38]	0.372
**RANTES/CCL5**	3.36 [3.28, 3.44]	3.34 [3.28, 3.42]	3.35 [3.29, 3.38]	3.40 [3.16, 3.62]	3.51 [3.43, 3.62]	0.183
**TGF-α**	0.82 [0.48, 1.10]	0.43 [0.36, 0.99]	0.77 [0.50, 0.95]	0.91 [0.88, 0.96]	1.47 [1.15, 1.73]	0.096
**TNF**	1.75 [1.58, 1.96]	1.70 [1.60, 1.81]	1.68 [1.45, 1.90]	1.79 [1.61, 2.02]	2.21 [2.11, 2.37]	0.040
**Lymphotoxin-α**	1.18 [0.98, 1.69]	1.09 [0.72, 1.28]	1.16 [1.01, 1.32]	1.23 [0.96, 1.67]	1.73 [1.70, 1.88]	0.055
**VEGF-A**	2.54 [2.16, 2.79]	1.94 [1.65, 2.38]	2.64 [2.38, 2.81]	2.56 [2.21, 2.86]	2.87 [2.69, 3.06]	0.038

Legend: [a]Mediator concentrations presented as $\log_{10}$ pg/ml with median and interquartile range; [b]Reflects results of Kruskal-Wallis H test; [c]Large proportion of values below the lower limit of quantification (see Supplemental Table 15).

Abbreviation: PLWH: persons living with HIV

Supplemental Table 5: Abundance of immune cell populations as inferred from CIBERTSORTx digital cytometry deconvolution, stratified by COVID-19 severity.

Immune cell population[a]	All patients N=100	Non-severe N=84	Severe N=16	p-value[b]
Naïve B cells	3.88 [2.57, 5.50]	3.82 [2.26, 5.26]	4.58 [3.26, 5.99]	0.229
Memory B cells	0.00 [0.00, 0.20]	0.00 [0.00, 0.20]	0.00 [0.00, 0.23]	0.923
Plasma cells	0.49 [0.26, 0.96]	0.44 [0.26, 0.75]	1.41 [0.72, 1.86]	0.001
$CD8^+$ T cells	2.05 [0.00, 4.02]	2.15 [0.21, 4.38]	0.00 [0.00, 2.79]	0.028
Naïve $CD4^+$ T cells	8.98 [5.84, 12.52]	8.98 [5.84, 12.82]	8.81 [6.94, 11.69]	0.645
Resting memory $CD4^+$ T cells	14.81 [11.82, 17.12]	14.92 [12.33, 17.16]	13.31 [5.52, 16.16]	0.094
Activated memory $CD4^+$ T cells	0.00 [0.00, 0.70]	0.00 [0.00, 0.66]	0.37 [0.00, 2.32]	0.104
Resting NK-cells	11.78 [9.73, 14.64]	12.04 [9.87, 14.79]	9.81 [8.29, 12.57]	0.055
Monocytes	8.80 [6.36, 11.12]	8.47 [6.32, 10.21]	11.35 [8.28, 13.99]	0.043
Activated dendritic cells	0.00 [0.00, 0.07]	0.00 [0.00, 0.07]	0.00 [0.00, 0.07]	0.604
Resting mast cells	0.40 [0.11, 0.72]	0.36 [0.16, 0.65]	0.64 [0.00, 1.30]	0.305
Neutrophils	10.83 [7.71, 18.77]	10.03 [6.96, 14.80]	50.67 [16.23, 66.67]	<0.001

Legend: [a]Eosinophils omitted from table given very low inferred absolute abundance across groups. [b]Reflects result of unadjusted two-sided Wilcoxon rank-sum test.

Supplemental Table 6: Association between COVID-19 severity and immune mediator concentrations among patients enrolled ≤7 days from symptom onset.

Mediator[a]	All patients N=85	Mild-moderate COVID-19 N=71	Severe COVID-19 N=14	Nominal p-value[b]	BH-adjusted p-value[b]
**sCD40L**	3.72 [3.39, 3.95]	3.71 [3.35, 3.93]	3.79 [3.40, 3.99]	0.355	0.604
**EGF**	2.10 [1.85, 2.42]	2.06 [1.83, 2.40]	2.35 [2.22, 2.64]	0.005	0.034
**Eotaxin**	1.49 [1.28, 1.66]	1.50 [1.30, 1.65]	1.36 [1.19, 1.69]	0.448	0.652
**FGF-2**	1.78 [1.50, 2.04]	1.72 [1.47, 2.03]	1.91 [1.74, 2.07]	0.248	0.496
**FLT-3L**	1.35 [1.11, 1.64]	1.35 [1.11, 1.65]	1.36 [1.18, 1.47]	0.887	0.959
**Fractalkine**	2.09 [1.92, 2.34]	2.04 [1.90, 2.31]	2.18 [2.04, 2.51]	0.155	0.384
**G-CSF[c]**	-0.18 [-0.18, 1.49]	-0.18 [-0.18, 1.46]	-0.18 [-0.18, 1.50]	0.939	0.959
**GM-CSF[c]**	-1.00 [-1.00, -1.00]	-1.00 [-1.00, -1.00]	-1.00 [-1.00, -1.00]	0.591	0.727
**GROα/CXCL1**	2.01 [1.77, 2.19]	2.00 [1.75, 2.13]	2.12 [1.94, 2.26]	0.160	0.384
**IFN-α2**	1.80 [1.61, 2.06]	1.78 [1.57, 2.05]	1.86 [1.72, 2.18]	0.171	0.391
**IFN-γ**	-0.08 [-1.40, 0.83]	-0.14 [-1.40, 0.84]	0.20 [-1.40, 0.65]	1.000	1.000
**IL-1α**	1.41 [1.10, 1.77]	1.34 [1.08, 1.77]	1.49 [1.28, 1.71]	0.216	0.451
**IL-1β**	1.45 [1.04, 1.75]	1.41 [1.03, 1.69]	1.76 [1.41, 1.85]	0.044	0.162
**IL-1Ra**	0.85 [0.57, 1.29]	0.78 [0.50, 1.14]	1.31 [1.13, 1.49]	0.002	0.016
**IL-2**	0.06 [-0.57, 0.51]	0.06 [-0.57, 0.51]	0.02 [-1.37, 0.50]	0.758	0.910
**IL-3[c]**	-2.00 [-2.00, -2.00]	-2.00 [-2.00, -2.00]	-2.00 [-2.00, -2.00]	0.578	0.728
**IL-4**	0.11 [-0.30, 0.37]	0.08 [-0.39, 0.36]	0.22 [0.11, 0.47]	0.104	0.293
**IL-5**	0.74 [0.29, 1.03]	0.81 [0.43, 1.03]	0.35 [0.07, 0.91]	0.062	0.213
**IL-6**	0.46 [0.08, 0.80]	0.38 [0.03, 0.71]	0.64 [0.53, 1.69]	0.017	0.082
**IL-7**	0.78 [0.58, 1.07]	0.75 [0.45, 0.95]	1.09 [0.88, 1.46]	0.001	0.016
**IL-8**	1.67 [1.21, 2.19]	1.70 [1.19, 2.11]	1.58 [1.41, 2.39]	0.441	0.652
**IL-9**	0.32 [-0.33, 0.79]	0.42 [-0.33, 0.79]	0.13 [-0.33, 0.93]	0.937	0.959
**IL-10**	0.62 [0.20, 1.19]	0.58 [0.23, 1.06]	0.98 [0.27, 1.50]	0.286	0.528
**IL-12p40**	1.68 [1.36, 1.95]	1.72 [1.48, 2.04]	1.31 [0.08, 1.65]	0.010	0.060
**IL-12p70**	0.44 [0.35, 0.63]	0.44 [0.34, 0.61]	0.41 [0.35, 0.85]	0.896	0.959
**IL-13**	1.73 [1.56, 2.09]	1.73 [1.55, 2.05]	1.74 [1.60, 2.15]	0.845	0.959
**IL-15**	1.26 [1.03, 1.57]	1.23 [1.02, 1.45]	1.69 [1.41, 1.87]	0.001	0.016
**IL-17A**	0.92 [0.56, 1.34]	0.84 [0.52, 1.24]	1.15 [0.72, 1.54]	0.110	0.293
**IL-17E/IL-25**	2.51 [2.20, 2.74]	2.49 [2.17, 2.74]	2.59 [2.33, 2.78]	0.434	0.652
**IL-17F**	0.48 [-0.25, 1.21]	0.34 [-0.25, 1.19]	1.14 [0.85, 1.41]	0.030	0.131
**IL-18**	1.71 [1.47, 1.93]	1.71 [1.44, 1.92]	1.70 [1.51, 1.98]	0.822	0.959
**IL-22**	1.69 [0.84, 1.93]	1.63 [0.84, 1.88]	1.74 [0.93, 2.05]	0.587	0.727
**IL-27**	3.21 [2.91, 3.41]	3.22 [2.92, 3.40]	3.19 [2.91, 3.47]	0.929	0.959
**IP-10/CXCL10**	2.42 [2.05, 2.80]	2.35 [2.02, 2.66]	3.10 [2.50, 3.63]	0.002	0.016
**MCP-1**	2.35 [2.14, 2.49]	2.36 [2.17, 2.49]	2.27 [2.01, 2.48]	0.477	0.673
**MCP-3**	1.17 [1.06, 1.44]	1.16 [1.04, 1.36]	1.35 [1.15, 1.58]	0.110	0.293
**M-CSF**	2.11 [1.86, 2.39]	2.10 [1.78, 2.39]	2.19 [2.00, 2.34]	0.365	0.604
**MDC**	3.08 [2.88, 3.22]	3.13 [2.93, 3.23]	2.88 [2.58, 3.06]	0.002	0.016
**MIG/CXCL9**	3.45 [3.26, 3.66]	3.44 [3.25, 3.60]	3.49 [3.35, 3.83]	0.201	0.439
**MIP-1α/CCL3**	1.56 [1.39, 1.95]	1.56 [1.39, 1.87]	1.64 [1.39, 2.39]	0.562	0.727
**MIP-1β/CCL4**	1.83 [1.64, 2.00]	1.80 [1.65, 1.99]	1.95 [1.68, 2.03]	0.303	0.539
**PDGF-AA**	3.51 [3.38, 3.68]	3.50 [3.38, 3.69]	3.57 [3.42, 3.65]	0.530	0.727
**PDGF-AB/BB**	4.46 [4.41, 4.52]	4.47 [4.42, 4.52]	4.42 [4.40, 4.46]	0.037	0.148
**RANTES**	3.36 [3.25, 3.46]	3.36 [3.25, 3.46]	3.34 [3.18, 3.44]	0.427	0.652
**TGF-α**	0.93 [0.59, 1.31]	0.88 [0.56, 1.20]	1.49 [1.12, 1.64]	<0.001	0.016
**TNF**	1.79 [1.60, 2.00]	1.78 [1.59, 1.94]	1.84 [1.73, 2.11]	0.260	0.499
**LT-α**	1.24 [1.02, 1.51]	1.20 [0.97, 1.46]	1.43 [1.25, 1.64]	0.067	0.214
**VEGF-A**	2.52 [2.18, 2.75]	2.42 [2.15, 2.73]	2.65 [2.55, 2.88]	0.013	0.069

Legend: [a]Mediator concentrations presented as $\log_{10}$ pg/ml with median and interquartile range; [b]Reflects results of two-sided Wilcoxon rank-sum test, unadjusted (nominal) and adjusted using Benjamini-Hochberg method; [c]Large proportion of values below the lower limit of quantification (see Supplemental Table 15).

Abbreviations: BH: Benjamini-Hochberg

Supplemental Table 7: Immune mediator concentrations stratified by SARS-CoV-2 variant-driven pandemic phase at time of hospitalization.

Mediator[a]	All patients N=306	Varied A/B Lineages N=97	A.23/A.23.1 N=141	Delta (B.1.617.2/AY) N=68	p-value[b]
**sCD40L**	3.77 [3.38, 3.99]	3.65 [3.40, 3.84]	3.70 [2.33, 3.96]	4.00 [3.86, 4.10]	<0.001
**EGF**	2.28 [1.95, 2.57]	2.10 [1.83, 2.35]	2.36 [2.01, 2.66]	2.45 [2.13, 2.61]	<0.001
**Eotaxin**	1.47 [1.24, 1.62]	1.51 [1.37, 1.62]	1.48 [1.21, 1.62]	1.43 [1.16, 1.60]	0.092
**FGF-2**	1.81 [1.51, 2.11]	1.71 [1.42, 2.10]	1.82 [1.57, 2.09]	1.89 [1.67, 2.17]	0.030
**FLT-3L**	1.38 [1.11, 1.62]	1.27 [1.08, 1.52]	1.48 [1.17, 1.72]	1.34 [1.10, 1.60]	0.005
**Fractalkine**	2.09 [1.95, 2.29]	2.04 [1.91, 2.29]	2.08 [1.96, 2.27]	2.18 [2.05, 2.47]	0.010
**G-CSF[c]**	-0.18 [-0.18, 1.30]	-0.18 [-0.18, 1.16]	-0.18 [-0.18, 1.26]	1.03 [-0.18, 1.53]	0.003
**GM-CSF[c]**	-1.00 [-1.00, -1.00]	-1.00 [-1.00, -1.00]	-1.00 [-1.00, -1.00]	-1.00 [-1.00, -1.00]	0.643
**GROα/CXCL1**	2.01 [1.83, 2.17]	1.97 [1.78, 2.11]	2.02 [1.83, 2.19]	2.03 [1.92, 2.18]	0.119
**IFN-α2**	1.82 [1.61, 2.04]	1.75 [1.46, 2.04]	1.84 [1.65, 1.98]	1.82 [1.66, 2.07]	0.180
**IFN-γ**	-0.08 [-1.40, 0.69]	-0.39 [-1.40, 0.59]	-0.09 [-1.40, 0.66]	0.16 [-1.40, 0.91]	0.130
**IL-1α**	1.41 [1.15, 1.77]	1.41 [1.08, 1.77]	1.40 [1.18, 1.75]	1.44 [1.22, 1.82]	0.412
**IL-1β**	1.44 [1.09, 1.78]	1.35 [0.89, 1.75]	1.47 [1.14, 1.74]	1.49 [1.14, 2.04]	0.064
**IL-1Ra**	0.86 [0.54, 1.31]	0.67 [0.41, 1.10]	0.82 [0.54, 1.25]	1.31 [0.99, 1.68]	<0.001
**IL-2**	0.01 [-0.62, 0.53]	-0.10 [-0.92, 0.51]	0.00 [-0.57, 0.52]	0.20 [-0.40, 0.63]	0.183
**IL-3[c]**	-2.00 [-2.00, -2.00]	-2.00 [-2.00, -2.00]	-2.00 [-2.00, -1.30]	-2.00 [-2.00, -2.00]	0.027
**IL-4**	0.20 [-0.11, 0.46]	0.08 [-0.36, 0.35]	0.25 [-0.06, 0.47]	0.30 [0.05, 0.60]	0.001
**IL-5**	0.71 [0.28, 1.12]	0.68 [0.25, 1.18]	0.69 [0.30, 1.06]	0.90 [0.21, 1.23]	0.775
**IL-6**	0.49 [0.10, 1.13]	0.26 [-0.10, 0.55]	0.57 [0.12, 1.16]	1.00 [0.45, 1.68]	<0.001
**IL-7**	0.84 [0.51, 1.08]	0.68 [0.41, 0.93]	0.88 [0.53, 1.10]	1.01 [0.78, 1.32]	<0.001
**IL-8**	1.78 [1.26, 2.37]	1.53 [1.02, 2.08]	1.70 [1.27, 2.47]	2.13 [1.73, 2.76]	<0.001
**IL-9**	0.59 [-0.33, 0.94]	-0.33 [-0.33, 0.71]	0.66 [-0.33, 0.96]	0.74 [-0.06, 1.01]	0.001
**IL-10**	0.60 [0.02, 1.16]	0.34 [-0.34, 0.77]	0.50 [-0.14, 0.89]	1.35 [0.77, 1.58]	<0.001
**IL-12p40**	1.64 [1.27, 1.91]	1.62 [1.20, 1.92]	1.66 [1.32, 1.89]	1.62 [1.20, 1.92]	0.768
**IL-12p70**	0.42 [0.29, 0.62]	0.40 [0.22, 0.62]	0.42 [0.32, 0.62]	0.47 [0.26, 0.66]	0.581
**IL-13**	1.86 [1.65, 2.18]	1.75 [1.54, 2.00]	1.91 [1.72, 2.15]	2.01 [1.73, 2.33]	0.003
**IL-15**	1.34 [1.11, 1.63]	1.14 [0.94, 1.35]	1.36 [1.19, 1.56]	1.64 [1.35, 1.87]	<0.001
**IL-17A**	0.97 [0.57, 1.41]	0.91 [0.40, 1.39]	0.97 [0.57, 1.36]	1.03 [0.63, 1.47]	0.216
**IL-17E/IL-25**	2.55 [2.27, 2.77]	2.41 [2.12, 2.66]	2.62 [2.41, 2.82]	2.58 [2.29, 2.77]	0.006
**IL-17F**	0.83 [-0.25, 1.49]	0.23 [-0.25, 1.25]	0.93 [-0.25, 1.54]	1.09 [-0.01, 1.58]	0.005
**IL-18**	1.68 [1.41, 1.93]	1.58 [1.28, 1.85]	1.69 [1.39, 1.90]	1.83 [1.61, 2.12]	<0.001
**IL-22**	1.81 [1.35, 2.06]	1.62 [0.84, 1.89]	1.88 [1.49, 2.06]	1.93 [1.64, 2.21]	<0.001
**IL-27**	3.15 [2.89, 3.37]	3.07 [2.84, 3.29]	3.13 [2.83, 3.35]	3.34 [3.13, 3.55]	<0.001
**IP-10/CXCL10**	2.25 [1.96, 2.60]	2.21 [1.99, 2.53]	2.28 [1.96, 2.56]	2.42 [1.92, 3.32]	0.082
**MCP-1**	2.35 [2.19, 2.48]	2.31 [2.21, 2.44]	2.37 [2.19, 2.51]	2.31 [2.13, 2.47]	0.218
**MCP-3**	1.24 [1.08, 1.55]	1.12 [1.02, 1.36]	1.26 [1.13, 1.60]	1.35 [1.17, 1.63]	<0.001
**M-CSF**	2.14 [1.87, 2.42]	1.96 [1.73, 2.27]	2.21 [2.01, 2.53]	2.22 [1.88, 2.50]	<0.001
**MDC**	3.03 [2.85, 3.20]	3.07 [2.94, 3.18]	3.04 [2.83, 3.21]	2.92 [2.73, 3.16]	0.008
**MIG/CXCL9**	3.47 [3.24, 3.67]	3.40 [3.22, 3.59]	3.49 [3.26, 3.69]	3.55 [3.24, 3.82]	0.038
**MIP-1α/CCL3**	1.67 [1.45, 2.22]	1.49 [1.34, 1.87]	1.66 [1.48, 2.09]	1.87 [1.61, 2.83]	<0.001
**MIP-1β/CCL4**	1.83 [1.64, 2.07]	1.76 [1.64, 1.97]	1.82 [1.59, 2.03]	2.06 [1.83, 2.35]	<0.001
**PDGF-AA**	3.53 [3.39, 3.69]	3.47 [3.36, 3.60]	3.58 [3.43, 3.73]	3.54 [3.42, 3.68]	0.005
**PDGF-AB/BB**	4.47 [4.42, 4.52]	4.47 [4.41, 4.51]	4.49 [4.44, 4.54]	4.45 [4.41, 4.52]	0.025
**RANTES**	3.37 [3.23, 3.48]	3.34 [3.22, 3.44]	3.41 [3.28, 3.54]	3.33 [3.19, 3.46]	0.011
**TGF-α**	1.07 [0.76, 1.47]	0.76 [0.49, 1.07]	1.10 [0.86, 1.35]	1.53 [1.16, 1.78]	<0.001
**TNF**	1.81 [1.64, 2.03]	1.74 [1.63, 1.88]	1.78 [1.62, 1.98]	2.02 [1.78, 2.26]	<0.001
**LT-α**	1.37 [1.14, 1.65]	1.18 [0.99, 1.43]	1.40 [1.20, 1.66]	1.53 [1.28, 1.76]	<0.001
**VEGF-A**	2.57 [2.31, 2.82]	2.48 [2.16, 2.71]	2.58 [2.36, 2.86]	2.70 [2.47, 2.96]	<0.001

Legend: [a]Mediator concentrations presented as log_{10} pg/ml with median and interquartile range; [b]Reflects results of Kruskal-Wallis H test; [c]Large proportion of values below the lower limit of quantification (see Supplemental Table 15).

Supplemental Table 8: Association between immune mediators and Delta phase COVID-19 (N=306).

Mediator	Delta phase β (95% CI)[a]	Nominal p-value	BH-adjusted p-value
sCD40L	0.552 (0.369 to 0.735)	<0.001	0.004364
EGF	0.143 (0.001 to 0.285)	0.049	0.1272
Eotaxin	-0.020 (-0.107 to 0.067)	0.655	0.731163
FGF-2	0.086 (-0.059 to 0.231)	0.242	0.37471
FLT-3L	-0.120 (-0.242 to 0.001)	0.052	0.1272
Fractalkine	0.107 (0.020 to 0.193)	0.016	0.048
G-CSF[b]	0.348 (0.111 to 0.585)	0.004	0.016
GM-CSF[b]	0.030 (-0.206 to 0.267)	0.801	0.873818
GROα/CXCL1	0.061 (-0.016 to 0.138)	0.122	0.254609
IFN-α2	-0.002 (-0.137 to 0.134)	0.981	0.981
IFN-γ	0.156 (-0.141 to 0.452)	0.302	0.419657
IL-1α	0.074 (-0.084 to 0.231)	0.358	0.464432
IL-1β	0.091 (-0.069 to 0.250)	0.266	0.399
IL-1Ra	0.397 (0.235 to 0.558)	<0.001	0.004364
IL-2	0.114 (-0.151 to 0.378)	0.398	0.489846
IL-3[b]	-0.055 (-0.264 to 0.155)	0.607	0.693714
IL-4	0.114 (-0.066 to 0.294)	0.213	0.3408
IL-5	0.115 (-0.052 to 0.283)	0.176	0.309517
IL-6	0.471 (0.283 to 0.659)	<0.001	0.004364
IL-7	0.106 (-0.024 to 0.237)	0.110	0.24
IL-8	0.487 (0.284 to 0.689)	<0.001	0.004364
IL-9	0.134 (-0.045 to 0.313)	0.141	0.282
IL-10	0.903 (0.641 to 1.166)	<0.001	0.004364
IL-12p40	0.004 (-0.191 to 0.199)	0.968	0.981
IL-12p70	-0.066 (-0.200 to 0.067)	0.329	0.438667
IL-13	0.093 (-0.035 to 0.221)	0.154	0.289846
IL-15	0.226 (0.108 to 0.344)	<0.001	0.004364
IL-17A	0.110 (-0.088 to 0.309)	0.275	0.4
IL-17E/IL-25	-0.107 (-0.265 to 0.052)	0.187	0.309517
IL-17F	0.089 (-0.164 to 0.342)	0.490	0.588
IL-18	0.193 (0.060 to 0.326	0.005	0.018462
IL-22	0.143 (-0.004 to 0.290)	0.056	0.128
IL-27	0.243 (0.143 to 0.342)	<0.001	0.004364
IP-10/CXCL10	0.230 (0.051 to 0.409)	0.012	0.0384
MCP-1	-0.044 (-0.108 to 0.021)	0.185	0.309517
MCP-3	0.060 (-0.055 to 0.174)	0.306	0.419657
M-CSF	0.010 (-0.168 to 0.189)	0.908	0.947478
MDC	-0.087 (-0.159 to -0.015)	0.018	0.050824
MIG/CXCL9	0.029 (-0.062 to 0.119)	0.534	0.625171
MIP-1α/CCL3	0.372 (0.200 to 0.544)	<0.001	0.004364
MIP-1β/CCL4	0.281 (0.190 to 0.373)	<0.001	0.004364
PDGF-AA	-0.006 (-0.079 to 0.066)	0.861	0.9184
PDGF-AB/BB	-0.013 (-0.041 to 0.016)	0.380	0.48

RANTES	-0.049 (-0.116 to 0.019)	0.157	0.289846
TGF-α	0.383 (0.248 to 0.519)	<0.001	0.004364
TNF	0.172 (0.081 to 0.264)	<0.001	0.004364
LT-α	0.145 (0.036 to 0.254)	0.009	0.030857
VEGF-A	0.122 (-0.002 to 0.245)	0.053	0.1272

Legend: [a]β reflects regression coefficient for Delta phase (hospitalization during Delta phase=1, hospitalization during either two prior phases=0) in a linear regression model with each log_{10} mediator concentration as dependent variable and age (continuous), sex (binary), HIV status (binary; 4 patients not known to be living with HIV but with missing rapid diagnostic tests analyzed as HIV negative), exposure to corticosteroids (binary), and WHO clinical severity (categorical) as independent co-variables; p-values reflect two-sided Wald test of mediator regression coefficient, adjusted using Benjamini-Hochberg method; [b]Large proportion of values below the lower limit of quantification (see Supplemental Table 15).

Abbreviations: BH: Benjamini-Hochberg, CI: Confidence interval

Supplemental Table 9: Association between inferred immune cell abundance and Delta phase COVID-19 (N=100).

Immune cell population	Delta phase β (95% CI)[a]	p-value
Naïve B cells	0.153 (-0.143 to 0.449)	0.308
Memory B cells	-0.695 (-1.717 to 0.326)	0.180
Plasma cells	-0.023 (-0.635 to 0.590)	0.942
$CD8^+$ T cells	-1.297 (-2.386 to -0.207)	0.020
Naïve $CD4^+$ T cells	-0.263 (-0.581 to 0.056	0.105
Resting memory $CD4^+$ T cells	-0.087 (-0.187 to 0.013)	0.088
Activated memory $CD4^+$ T cells	0.890 (-0.191 to 1.972)	0.105
Resting NK-cells	-0.096 (-0.168 to -0.024)	0.009
Monocytes	0.061 (-0.039 to 0.160)	0.228
Activated dendritic cells	-0.267 (-1.146 to 0.611)	0.547
Resting mast cells	0.211 (-0.716 to 1.139	0.652
Eosinophils	-0.336 (-1.113 to 0.441)	0.392
Neutrophils	0.231 (0.067 to 0.39)	0.006

Legend: [a]β reflects regression coefficient for Delta phase (hospitalization during Delta phase=1, hospitalization during either two prior phases=0) in a linear regression model with each log_{10} immune cell abundance as dependent variable and age (continuous), sex (binary), HIV status (binary; 2 patients not known to be living with HIV but with missing rapid diagnostic tests analyzed as HIV negative), exposure to corticosteroids (binary), and WHO clinical severity (categorical) as independent co-variables; p-values reflect unadjusted two-sided Wald test of mediator regression coefficient.

Abbreviations: BH: Benjamini-Hochberg, CI: Confidence interval

Supplemental Table 10: Optimal mediator-derived cluster partition (*k*) among patients with symptomatic COVID-19 (N=240).

**Index**	**Optimal number of clusters (*k*)**
KL	2
CH	2
Hartigan	4
CCC	2
Scott	7
Marriot	4
TrCovW	3
TraceW	4
Friedman	3
Rubin	7
Cindex	4
DB	2
Silhouette	2
Duda	2
PseudoT2	2
Beale	2
Ratkowsky	2
Ball	3
PtBiserial	4
McClain	2
Dunn	7
SDindex	3
SDbw	12

Supplemental Table 11: Immune mediator concentrations stratified by cluster-derived COVID-19 Response Signatures (CRS).

Mediator[a]	All patients N=240	CRS-1 N=142	CRS-2 N=98	P-value[b]
**sCD40L**	3.80 [3.40, 4.00]	3.66 [3.18, 3.89]	3.96 [3.74, 4.07]	<0.001
**EGF**	2.27 [1.95, 2.56]	2.13 [1.83, 2.54]	2.37 [2.11, 2.58]	0.014
**Eotaxin**	1.46 [1.20, 1.64]	1.45 [1.19, 1.61]	1.48 [1.23, 1.69]	0.226
**FGF-2**	1.78 [1.54, 2.11]	1.64 [1.42, 1.87]	2.08 [1.82, 2.37]	<0.001
**FLT-3L**	1.38 [1.11, 1.64]	1.25 [1.02, 1.51]	1.56 [1.29, 1.78]	<0.001
**Fractalkine**	2.08 [1.95, 2.32]	2.00 [1.90, 2.12]	2.29 [2.13, 2.67]	<0.001
**G-CSF[c]**	-0.18 [-0.18, 1.33]	-0.18 [-0.18, 0.99]	1.14 [-0.18, 1.66]	<0.001
**GM-CSF[c]**	-1.00 [-1.00, -1.00]	-1.00 [-1.00, -1.00]	-1.00 [-1.00, 0.32]	<0.001
**GRO-α/CXCL1**	2.01 [1.83, 2.19]	1.95 [1.76, 2.13]	2.08 [1.94, 2.22]	0.001
**IFN-α2**	1.83 [1.62, 2.04]	1.71 [1.53, 1.85]	2.02 [1.84, 2.34]	<0.001
**IFN-γ**	-0.03 [-1.40, 0.69]	-1.20 [-1.40, 0.05]	0.67 [0.10, 1.19]	<0.001
**IL-1a**	1.41 [1.14, 1.75]	1.27 [1.05, 1.52]	1.68 [1.31, 2.11]	<0.001
**IL-1β**	1.45 [1.11, 1.79]	1.31 [0.95, 1.55]	1.78 [1.46, 2.19]	<0.001
**IL-1Ra**	0.89 [0.55, 1.39]	0.67 [0.41, 0.92]	1.38 [0.96, 1.82]	<0.001
**IL-2**	0.01 [-0.57, 0.52]	-0.25 [-1.30, 0.12]	0.52 [0.05, 1.18]	<0.001
**IL-3[c]**	-2.00 [-2.00, -2.00]	-2.00 [-2.00, -2.00]	-2.00 [-2.00, -0.73]	<0.001
**IL-4**	0.21 [-0.08, 0.48]	0.05 [-0.32, 0.27]	0.45 [0.25, 0.63]	<0.001
**IL-5**	0.67 [0.25, 1.06]	0.60 [0.18, 0.98]	0.80 [0.38, 1.11]	0.020
**IL-6**	0.53 [0.11, 1.17]	0.29 [0.03, 0.67]	1.01 [0.49, 1.62]	<0.001
**IL-7**	0.85 [0.57, 1.14]	0.71 [0.43, 0.98]	1.06 [0.78, 1.42]	<0.001
**IL-8**	1.74 [1.27, 2.28]	1.61 [1.11, 2.10]	2.00 [1.50, 2.59]	<0.001
**IL-9**	0.59 [-0.33, 0.94]	0.19 [-0.33, 0.70]	0.86 [0.55, 1.26]	<0.001
**IL-10**	0.65 [0.16, 1.23]	0.53 [-0.17, 0.93]	0.92 [0.50, 1.55]	<0.001
**IL-12p40**	1.62 [1.26, 1.91]	1.53 [1.15, 1.73]	1.86 [1.54, 2.16]	<0.001
**IL-12p70**	0.42 [0.29, 0.62]	0.35 [0.23, 0.48]	0.60 [0.41, 1.09]	<0.001
**IL-13**	1.87 [1.66, 2.17]	1.73 [1.54, 1.92]	2.18 [1.96, 2.53]	<0.001
**IL-15**	1.35 [1.15, 1.66]	1.23 [1.03, 1.38]	1.65 [1.42, 1.96]	<0.001
**IL-17A**	0.97 [0.57, 1.38]	0.75 [0.40, 1.06]	1.33 [0.79, 1.78]	<0.001
**IL-17E/IL-25**	2.55 [2.30, 2.76]	2.41 [2.05, 2.60]	2.73 [2.57, 2.90]	<0.001
**IL-17F**	0.94 [-0.25, 1.48]	0.38 [-0.25, 1.23]	1.33 [0.77, 1.66]	<0.001
**IL-18**	1.71 [1.44, 1.94]	1.63 [1.40, 1.87]	1.80 [1.61, 2.09]	<0.001
**IL-22**	1.81 [1.26, 2.07]	1.61 [0.84, 1.83]	2.08 [1.90, 2.30]	<0.001
**IL-27**	3.17 [2.90, 3.38]	3.07 [2.82, 3.32]	3.31 [3.09, 3.50]	<0.001
**IP-10/CXCL10**	2.34 [2.02, 2.75]	2.24 [1.94, 2.54]	2.50 [2.21, 3.32]	<0.001
**MCP-1**	2.35 [2.18, 2.48]	2.31 [2.16, 2.44]	2.40 [2.24, 2.53]	0.006
**MCP-3**	1.25 [1.09, 1.58]	1.13 [1.03, 1.27]	1.59 [1.29, 1.95]	<0.001
**M-CSF**	2.16 [1.87, 2.42]	2.04 [1.69, 2.31]	2.32 [2.10, 2.62]	<0.001
**MDC**	3.03 [2.82, 3.18]	3.06 [2.87, 3.21]	2.98 [2.79, 3.16]	0.102
**MIG/CXCL9**	3.48 [3.25, 3.68]	3.39 [3.18, 3.61]	3.56 [3.36, 3.85]	<0.001
**MIP-1α/CCL3**	1.63 [1.44, 2.12]	1.50 [1.37, 1.69]	1.88 [1.65, 2.72]	<0.001
**MIP-1β/CCL4**	1.83 [1.63, 2.04]	1.74 [1.57, 1.97]	1.94 [1.78, 2.20]	<0.001
**PDGF-AA**	3.54 [3.40, 3.69]	3.51 [3.38, 3.65]	3.63 [3.44, 3.76]	0.006
**PDGF-AB/BB**	4.47 [4.42, 4.52]	4.47 [4.42, 4.53]	4.46 [4.42, 4.51]	0.291
**RANTES/CCL5**	3.38 [3.25, 3.49]	3.39 [3.28, 3.49]	3.35 [3.23, 3.48]	0.101
**TGF-α**	1.08 [0.77, 1.49]	0.92 [0.65, 1.20]	1.30 [1.01, 1.69]	<0.001
**TNF**	1.81 [1.65, 2.05]	1.72 [1.58, 1.82]	2.08 [1.88, 2.37]	<0.001
**Lymphotoxin-α**	1.38 [1.13, 1.67]	1.20 [1.00, 1.40]	1.72 [1.48, 1.87]	<0.001
**VEGF-A**	2.58 [2.33, 2.86]	2.49 [2.20, 2.71]	2.78 [2.51, 3.03]	<0.001

Legend: [a]Mediator concentrations presented as log_{10} pg/ml with median and interquartile range; [b]Reflects results of unadjusted two-sided Wilcoxon rank-sum test; [c]Large proportion of values below the lower limit of quantification (see Supplemental Table 15).

Supplemental Table 12: Association between mediator concentrations and HIV co-infection among patients with SARS-CoV-2 (N=302; 4 patients without definitive assessment of HIV status excluded).

Mediator	β for HIV co-infection (95% CI)[a]	Nominal p-value	BH-adjusted p-value
sCD40L	-0.038 (-0.295 to 0.219)	0.769	0.785362
EGF	-0.195 (-0.385 to -0.004)	0.045	0.1632
Eotaxin	0.029 (-0.086 to 0.144)	0.621	0.699273
FGF-2	-0.227 (-0.422 to -0.032)	0.023	0.1632
FLT-3L	-0.113 (-0.276 to 0.050)	0.175	0.343385
Fractalkine	-0.093 (-0.210 to 0.025)	0.122	0.266182
G-CSF[b]	-0.122 (-0.440 to 0.196)	0.450	0.568421
GM-CSF[b]	0.002 (-0.315 to 0.320)	0.990	0.99
GROα/CXCL1	-0.113 (-0.217 to -0.010)	0.032	0.1632
IFN-α2	-0.212 (-0.393 to -0.031)	0.022	0.1632
IFN-γ	-0.250 (-0.650 to 0.150)	0.219	0.3504
IL-1α	-0.125 (-0.336 to 0.087)	0.247	0.3705
IL-1β	-0.349 (-0.563 to -0.135)	0.001	0.048
IL-1Ra	-0.230 (-0.455 to -0.005)	0.045	0.1632
IL-2	-0.382 (-0.736 to -0.028)	0.034	0.1632
IL-3[b]	0.302 (-0.583 to -0.021)	0.035	0.1632
IL-4	-0.213 (-0.455 to 0.030)	0.085	0.214737
IL-5	0.235 (0.011 to 0.458)	0.040	0.1632
IL-6	-0.063 (-0.323 to 0.196)	0.630	0.699273
IL-7	-0.047 (-0.224 to 0.129)	0.597	0.698927
IL-8	-0.174 (-0.452 to 0.103)	0.218	0.3504
IL-9	-0.129 (-0.369 to 0.110)	0.288	0.406588
IL-10	-0.071 (-0.449 to 0.306)	0.710	0.757333
IL-12p40	-0.292 (-0.550 to -0.033)	0.027	0.1632
IL-12p70	-0.087 (-0.266 to 0.092	0.340	0.453333
IL-13	-0.138 (-0.309 to 0.034)	0.115	0.262857
IL-15	-0.158 (-0.322 to 0.005)	0.058	0.174
IL-17A	-0.275 (-0.542 to -0.008)	0.043	0.1632
IL-17E/IL-25	-0.173 (-0.386 to 0.040)	0.111	0.262857
IL-17F	-0.316 (-0.656 to 0.025)	0.069	0.194824
IL-18	-0.081 (-0.258 to 0.097)	0.371	0.481297
IL-22	-0.105 (-0.302 to 0.093)	0.299	0.410057
IL-27	-0.081 (-0.219 to 0.057)	0.247	0.3705
IP-10/CXCL10	0.078 (-0.163 to 0.318)	0.526	0.647385
MCP-1	0.065 (-0.021 to 0.152)	0.137	0.285913
MCP-3	0.023 (-0.130 to 0.176)	0.766	0.785362
M-CSF	-0.055 (-0.288 to 0.178)	0.641	0.699273
MDC	-0.054 (-0.150 to 0.043)	0.275	0.400
MIG/CXCL9	0.083 (-0.038 to 0.203)	0.180	0.343385
MIP-1α/CCL3	-0.284 (-0.519 to -0.050)	0.018	0.1632
MIP-1β/CCL4	-0.082 (-0.210 to 0.047	0.211	0.3504
PDGF-AA	-0.096 (-0.192 to 0.000)	0.051	0.1632
PDGF-AB/BB	-0.054 (-0.091 to -0.016	0.005	0.120
RANTES	0.026 (-0.064 to 0.116	0.572	0.6864

TGF-α	-0.197 (-0.394 to 0.000)	0.050	0.1632
TNF	-0.079 (-0.204 to 0.046)	0.216	0.3504
LT-α	-0.133 (-0.281 to 0.015)	0.079	0.210667
VEGF-A	-0.111 (-0.276 to 0.054)	0.186	0.343385

Legend: [a]β reflects regression coefficient for HIV co-infection (living with HIV=1, not living with HIV=0) in a linear regression model with each $\log_{10}$ mediator concentration as dependent variable and age (continuous), sex (binary), and WHO clinical severity (categorical) as independent co-variables; p-values reflect two-sided Wald test of mediator regression coefficient, adjusted using Benjamini-Hochberg method; [b]Large proportion of values below the lower limit of quantification (see Supplemental Table 15).

Abbreviations: BH: Benjamini-Hochberg, CI: confidence interval

Supplemental Table 13: Association between COVID-19 status and immune mediator concentrations among patients with COVID-19, influenza SARI, and non-influenza SARI in Uganda (N=292; 66 patients with asymptomatic SARS-CoV-2 infection excluded).

Mediator	COVID-19 β (95% CI)[a]	Nominal p-value	BH-adjusted p-value
sCD40L	0.074 (−0.149 to 0.297)	0.513	0.684
EGF	0.656 (0.477 to 0.836)	<0.001	0.0048
Eotaxin	0.193 (0.080 to 0.307)	<0.001	0.0048
FGF-2	0.067 (−0.109 to 0.243)	0.453	0.639529
FLT-3L	0.231 (0.081 to 0.382)	0.003	0.013091
Fractalkine	−0.006 (−0.117 to 0.105)	0.92	0.975652
G-CSF[b]	−0.179 (−0.488 to 0.130)	0.255	0.3825
GM-CSF[b]	0.006 (−0.295 to 0.308)	0.968	0.981
GROα/CXCL1	0.035 (−0.060 to 0.130)	0.47	0.644571
IFN-α2	0.106 (−0.069 to 0.281)	0.235	0.3824
IFN-γ	−0.289 (−0.657 to 0.078)	0.122	0.23424
IL-1α	0.009 (−0.179 to 0.196)	0.929	0.975652
IL-1β	−0.015 (−0.212 to 0.182)	0.884	0.975652
IL-1Ra	−0.197 (−0.411 to 0.017)	0.071	0.158609
IL-2	0.018 (−0.310 to 0.346)	0.914	0.975652
IL-3[b]	0.038 (−0.194 to 0.269)	0.75	0.923077
IL-4	0.215 (−0.012 to 0.442)	0.063	0.158609
IL-5	−0.114 (−0.306 to 0.077)	0.239	0.3824
IL-6	−0.290 (−0.543 to −0.037)	0.025	0.092308
IL-7	0.1 (−0.071 to 0.271)	0.25	0.3825
IL-8	0.483 (0.249 to 0.716)	<0.001	0.0048
IL-9	0.228 (0.013 to 0.444)	0.038	0.12
IL-10	−0.737 (−1.072 to −0.401)	<0.001	0.0048
IL-12p40	0.177 (−0.073 to 0.426)	0.165	0.304615
IL-12p70	−0.023 (−0.194 to 0.148)	0.79	0.948
IL-13	0.002 (−0.156 to 0.160)	0.981	0.981
IL-15	0.014 (−0.131 to 0.159)	0.851	0.975652
IL-17A	0.04 (−0.204 to 0.284)	0.747	0.923077
IL-17E/IL-25	0.201 (0.006 to 0.395)	0.043	0.121412
IL-17F	0.274 (−0.029 to 0.577)	0.076	0.158609
IL-18	−0.193 (−0.364 to −0.023)	0.027	0.092571
IL-22	0.112 (−0.071 to 0.295)	0.231	0.3824
IL-27	−0.359 (−0.491 to −0.228)	<0.001	0.0048
IP-10/CXCL10	−0.440 (−0.696 to −0.184)	<0.001	0.0048
MCP-1	0.004 (−0.082 to 0.089)	0.935	0.975652
MCP-3	0.124 (−0.012 to 0.260)	0.074	0.158609
M-CSF	0.507 (0.270 to 0.744)	<0.001	0.0048
MDC	0.073 (−0.012 to 0.157)	0.094	0.188
MIG/CXCL9	−0.337 (−0.455 to −0.218)	<0.001	0.0048
MIP-1α/CCL3	0.205 (−0.002 to 0.413)	0.052	0.138667
MIP-1β/CCL4	0.074 (−0.035 to 0.184)	0.184	0.327111
PDGF-AA	0.267 (0.175 to 0.359)	<0.001	0.0048
PDGF-AB/BB	0.082 (0.043 to 0.122)	<0.001	0.0048
RANTES/CCL5	0.102 (0.025 to 0.179)	0.01	0.04

TGF-α	0.179 (0.008 to 0.350)	0.04	0.12
TNF	−0.061 (−0.177 to 0.055)	0.303	0.440727
LT-α	−0.023 (−0.159 to 0.113)	0.738	0.923077
VEGF-A	0.147 (−0.012 to 0.305)	0.069	0.158609

Legend: [a]β reflects regression coefficient for COVID-19 status (binary; SARS-CoV-2 positive=1, influenza or non-influenza SARI=0) in a linear regression model with each $\log_{10}$ mediator concentration as dependent variable and age (continuous), sex (binary), HIV status (binary; 4 patients not known to be living with HIV but with missing rapid diagnostic tests analyzed as HIV negative) and WHO clinical severity status (categorical) as independent co-variables; p-values reflect two-sided Wald test of mediator regression coefficient, adjusted using Benjamini-Hochberg method; [b]Large proportion of values below the lower limit of quantification (see Supplemental Table 15).

Abbreviations: BH: Benjamini-Hochberg, CI: confidence interval

Supplemental Table 14: Immune mediator concentrations stratified by SARI etiology (N=292; 66 patients with asymptomatic SARS-CoV-2 infection excluded).

Mediator[a]	All patients N=292	COVID-19 N=240	Influenza SARI N=15	Non-influenza SARI N=37	p-value[b]
**sCD40L**	3.76 [3.39, 3.98]	3.80 [3.40, 4.00]	3.66 [3.26, 3.87]	3.68 [3.41, 3.85]	0.175
**EGF**	2.15 [1.78, 2.51]	2.27 [1.95, 2.56]	1.32 [0.24, 1.96]	1.70 [1.28, 2.07]	<0.001
**Eotaxin**	1.45 [1.18, 1.62]	1.46 [1.20, 1.64]	1.41 [1.21, 1.61]	1.33 [0.94, 1.48]	0.018
**FGF-2**	1.79 [1.55, 2.13]	1.78 [1.54, 2.11]	1.68 [1.60, 1.86]	1.86 [1.65, 2.31]	0.157
**FLT-3L**	1.35 [1.08, 1.61]	1.38 [1.11, 1.64]	1.20 [1.07, 1.44]	1.14 [0.79, 1.40]	0.003
**Fractalkine**	2.08 [1.94, 2.32]	2.08 [1.95, 2.32]	2.16 [2.05, 2.43]	2.06 [1.91, 2.32]	0.698
**G-CSF[c]**	-0.18 [-0.18, 1.40]	-0.18 [-0.18, 1.33]	1.88 [-0.18, 2.06]	-0.18 [-0.18, 1.32]	0.025
**GM-CSF[c]**	-1.00 [-1.00, -1.00]	-1.00 [-1.00, -1.00]	-1.00 [-1.00, -1.00]	-1.00 [-1.00, -1.00]	0.769
**GROα/CXCL1**	2.01 [1.83, 2.17]	2.01 [1.83, 2.19]	2.07 [1.93, 2.44]	1.98 [1.79, 2.08]	0.064
**IFN-α2**	1.83 [1.61, 2.07]	1.83 [1.62, 2.04]	1.72 [1.37, 2.06]	1.96 [1.67, 2.36]	0.256
**IFN-γ**	0.01 [-1.40, 0.79]	-0.03 [-1.40, 0.69]	0.30 [-0.78, 0.69]	0.31 [-1.40, 1.25]	0.183
**IL-1α**	1.41 [1.14, 1.77]	1.41 [1.14, 1.75]	1.35 [1.05, 1.68]	1.50 [1.25, 2.20]	0.358
**IL-1β**	1.44 [1.11, 1.82]	1.45 [1.11, 1.79]	1.20 [0.82, 1.64]	1.72 [1.14, 2.19]	0.028
**IL-1Ra**	0.93 [0.56, 1.46]	0.89 [0.55, 1.39]	1.25 [0.76, 1.75]	1.08 [0.71, 1.76]	0.116
**IL-2**	0.02 [-0.57, 0.55]	0.01 [-0.57, 0.52]	-0.32 [-1.70, 0.23]	0.36 [-0.25, 1.26]	0.015
**IL-3[c]**	-2.00 [-2.00, -2.00]	-2.00 [-2.00, -2.00]	-2.00 [-2.00, -2.00]	-2.00 [-2.00, -1.52]	0.512
**IL-4**	0.19 [-0.12, 0.48]	0.21 [-0.08, 0.48]	0.10 [-0.39, 0.48]	0.11 [-0.37, 0.42]	0.359
**IL-5**	0.70 [0.27, 1.08]	0.67 [0.25, 1.06]	0.69 [0.50, 1.00]	0.84 [0.46, 1.24]	0.237
**IL-6**	0.57 [0.12, 1.22]	0.53 [0.11, 1.17]	1.29 [0.90, 1.48]	0.70 [0.13, 1.18]	0.017
**IL-7**	0.85 [0.58, 1.13]	0.85 [0.57, 1.14]	0.87 [0.69, 1.06]	0.84 [0.60, 1.11]	0.989
**IL-8**	1.64 [1.20, 2.19]	1.74 [1.27, 2.28]	1.28 [0.91, 1.57]	1.36 [1.07, 1.67]	<0.001
**IL-9**	0.56 [-0.33, 0.90]	0.59 [-0.33, 0.94]	0.53 [-0.33, 0.77]	-0.23 [-0.33, 0.89]	0.162
**IL-10**	0.74 [0.26, 1.38]	0.65 [0.16, 1.23]	1.11 [0.72, 1.54]	1.34 [0.79, 1.70]	<0.001
**IL-12p40**	1.62 [1.25, 1.92]	1.62 [1.26, 1.91]	1.41 [1.12, 2.13]	1.63 [0.79, 2.09]	0.953
**IL-12p70**	0.42 [0.29, 0.63]	0.42 [0.29, 0.62]	0.48 [0.23, 0.59]	0.39 [0.18, 0.74]	0.879
**IL-13**	1.87 [1.65, 2.17]	1.87 [1.66, 2.17]	1.77 [1.39, 1.97]	1.89 [1.69, 2.19]	0.292
**IL-15**	1.36 [1.12, 1.65]	1.35 [1.15, 1.66]	1.47 [1.42, 1.62]	1.27 [1.03, 1.61]	0.387
**IL-17A**	0.97 [0.56, 1.42]	0.97 [0.57, 1.38]	0.72 [0.33, 1.07]	1.33 [0.63, 1.81]	0.029
**IL-17E/IL-25**	2.53 [2.28, 2.75]	2.55 [2.30, 2.76]	2.49 [2.28, 2.62]	2.45 [1.97, 2.76]	0.327
**IL-17F**	0.87 [-0.25, 1.48]	0.94 [-0.25, 1.48]	0.87 [-0.25, 1.19]	0.07 [-0.25, 1.60]	0.231
**IL-18**	1.72 [1.46, 1.99]	1.71 [1.44, 1.94]	1.88 [1.72, 2.18]	1.92 [1.48, 2.24]	0.021
**IL-22**	1.79 [1.21, 2.04]	1.81 [1.26, 2.07]	1.50 [0.84, 1.97]	1.74 [0.84, 1.97]	0.326
**IL-27**	3.22 [2.94, 3.46]	3.17 [2.90, 3.38]	3.68 [3.30, 3.95]	3.49 [3.28, 3.77]	<0.001
**IP-10/CXCL10**	2.38 [2.04, 2.87]	2.34 [2.02, 2.75]	3.34 [2.57, 3.78]	2.43 [2.22, 3.11]	0.007
**MCP-1**	2.34 [2.18, 2.49]	2.35 [2.18, 2.48]	2.54 [2.35, 2.63]	2.27 [2.09, 2.38]	0.006
**MCP-3**	1.23 [1.09, 1.53]	1.25 [1.09, 1.58]	1.15 [1.04, 1.23]	1.20 [1.00, 1.36]	0.101
**M-CSF**	2.12 [1.83, 2.40]	2.16 [1.87, 2.42]	2.12 [1.91, 2.51]	1.87 [1.17, 2.14]	0.001
**MDC**	3.01 [2.80, 3.17]	3.03 [2.82, 3.18]	2.92 [2.79, 3.25]	2.95 [2.79, 3.06]	0.245
**MIG/CXCL9**	3.53 [3.28, 3.76]	3.48 [3.25, 3.68]	3.80 [3.53, 4.04]	3.76 [3.55, 4.14]	<0.001
**MIP-1α/CCL3**	1.60 [1.43, 2.05]	1.63 [1.44, 2.12]	1.46 [1.40, 1.53]	1.55 [1.40, 2.09]	0.019
**MIP-1β/CCL4**	1.82 [1.62, 2.02]	1.83 [1.63, 2.04]	1.68 [1.56, 1.95]	1.85 [1.58, 1.95]	0.160
**PDGF-AA**	3.50 [3.34, 3.68]	3.54 [3.40, 3.69]	3.33 [3.17, 3.66]	3.30 [3.07, 3.42]	<0.001
**PDGF-AB/BB**	4.46 [4.40, 4.52]	4.47 [4.42, 4.52]	4.42 [4.36, 4.46]	4.39 [4.35, 4.44]	<0.001
**RANTES**	3.36 [3.23, 3.47]	3.38 [3.25, 3.49]	3.26 [3.16, 3.49]	3.26 [3.16, 3.35]	0.001
**TGF-α**	1.06 [0.74, 1.40]	1.08 [0.77, 1.49]	0.80 [0.63, 1.12]	0.96 [0.64, 1.17]	0.043
**TNF**	1.83 [1.67, 2.09]	1.81 [1.65, 2.05]	1.95 [1.80, 2.18]	1.93 [1.69, 2.15]	0.210
**LT-α**	1.38 [1.13, 1.68]	1.38 [1.13, 1.67]	1.21 [1.07, 1.64]	1.43 [1.18, 1.72]	0.589
**VEGF-A**	2.56 [2.31, 2.84]	2.58 [2.33, 2.86]	2.37 [2.17, 2.90]	2.49 [2.25, 2.71]	0.189

Legend: [a]Mediator concentrations presented as log_{10} pg/ml with median and interquartile range; [b]Reflects results of Kruskal-Wallis H test; [c]Large proportion of values below the lower limit of quantification (see Supplemental Table 15).

Supplemental Table 15: Proportions of immune mediator concentrations outside the limits of quantification in multiplexed immunoassay

Mediator	Proportion of values below lower limit of quantification (%)	Proportion of values above upper limit of quantification (%)
sCD40L	0	0
EGF	2.2	0
Eotaxin	0	0
FGF-2	2.2	0
FLT-3L	1.1	0
Fractalkine	0	0
G-CSF	55.2	0
GM-CSF	89.1	0
GROα/CXCL1	0	0
IFN-α2	4.2	0
IFN-γ	32.2	0
IL-1α	0	0
IL-1β	0.8	0
IL-1Ra	0	0
IL-2	14	0
IL-3	75.6	0
IL-4	9	0
IL-5	0	0
IL-6	0	0
IL-7	1.1	0
IL-8	0	0
IL-9	35	0
IL-10	9	0
IL-12p40	8.4	0
IL-12p70	0	0
IL-13	2	0
IL-15	0	0
IL-17A	2.2	0
IL-17E/IL-25	8.4	0
IL-17F	35.6	0
IL-18	1.4	0
IL-22	22.4	0
IL-27	0	0
IP-10/CXCL10	0	0
MCP-1	0	0
MCP-3	0.3	0
M-CSF	4.5	0
MDC	0	0
MIG/CXCL9	0	0
MIP-1α/CCL3	0.3	0
MIP-1β/CCL4	0	0
PDGF-AA	0	0
PDGF-AB/BB	0	0.3
RANTES	0	0
TGF-α	0	0
TNF	0	0
LT-α	0	0
VEGF-A	0	0

Supplemental Table 16: Missing clinical and laboratory variables and imputation approach

Variable	Proportion of symptomatic COVID-19 patients with missing data (%)	Approach to imputation[a]
Age	0.0	N/A
Sex	0.0	N/A
Karnofsky Performance Status	0.0	N/A
Symptom duration at enrollment	14.6	Multiple imputation via chained equations and PMM
Temperature	2.5	Multiple imputation via chained equations and PMM
Heart rate	0.42	Multiple imputation via chained equations and PMM
Respiratory rate	5.4	Multiple imputation via chained equations and PMM
Systolic blood pressure	0.42	Multiple imputation via chained equations and PMM
Peripheral oxygen saturation	0.0	N/A
Whole-blood lactate concentration	10.0	Multiple imputation via chained equations and PMM
Hemoglobin concentration	62.5	Multiple imputation via chained equations and PMM
White blood cell count	62.9	Multiple imputation via chained equations and PMM
Platelet count	62.9	Multiple imputation via chained equations and PMM
Duration of hospitalization[b]	4.2	Multiple imputation via chained equations and PMM

Legend: [a]Multiple imputation via chained equations and PMM performed using all clinical and laboratory variables as predictors; [b]Hospital outcome (death or transfer vs. discharge alive) also used as predictor.

Abbreviations: PMM: predictive mean matching

Supplemental Figures

Supplemental Figure 1: Flow diagram for adult patients prospectively enrolled in RESERVE-U-C19. Figure created in Biorender.

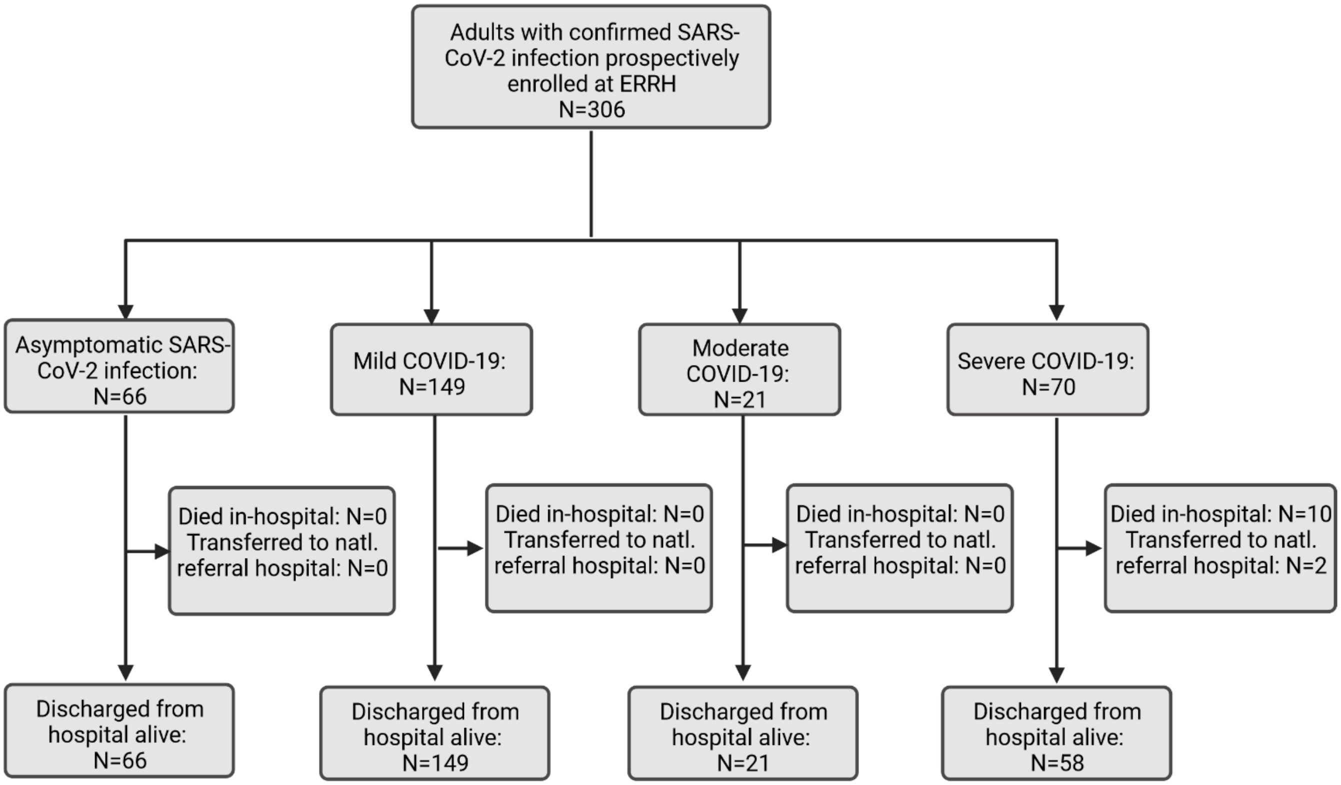

Supplemental Figure 2: Immune mediator concentrations stratified by COVID-19 severity in persons living with HIV (N=33).

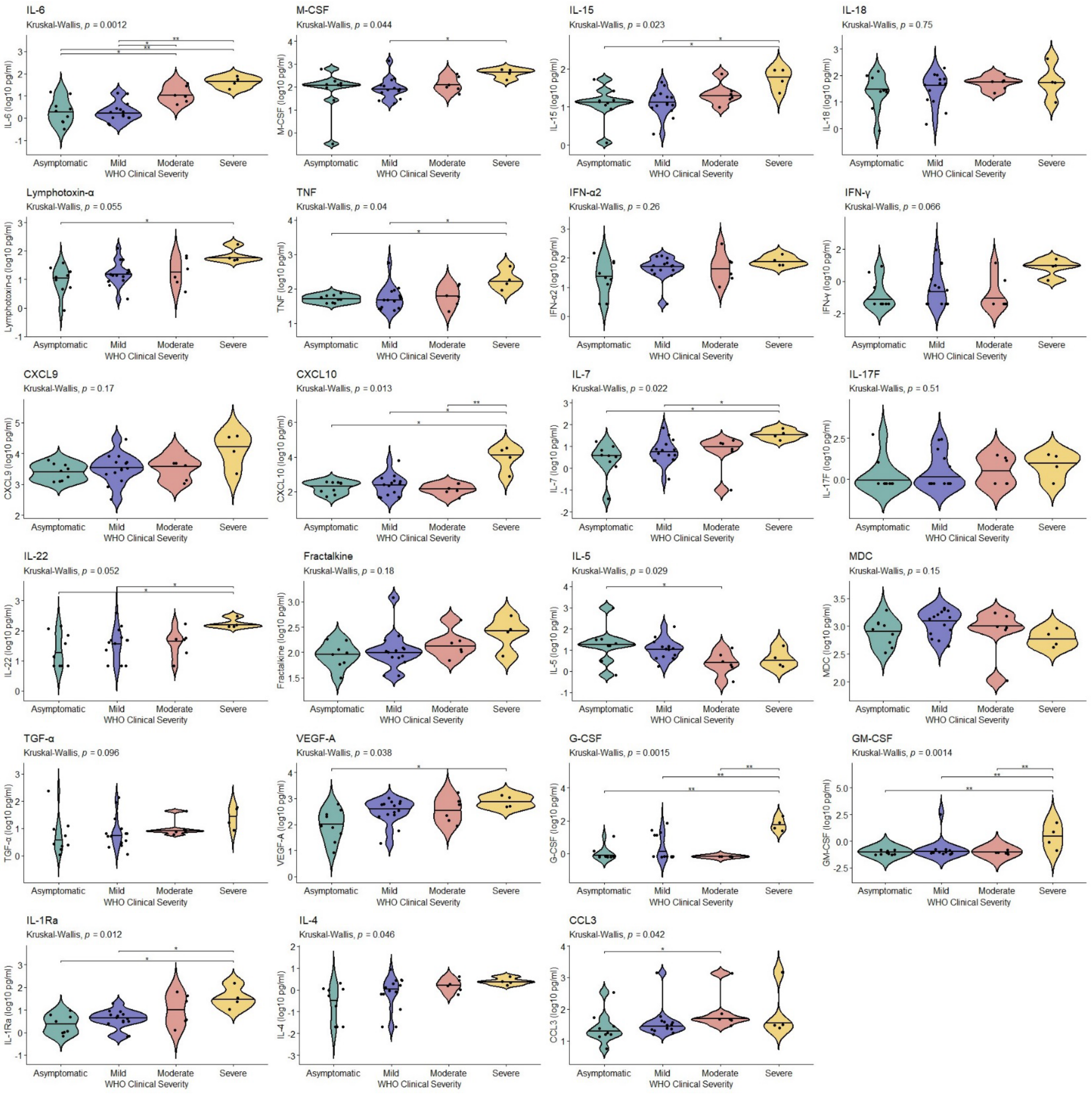

Mediators significantly associated with escalating COVID-19 severity in general study population and representative of key immunopathological domains presented along with other mediators significantly associated with COVID-19 severity in persons living with HIV (p≤0.05 in Kruskal-Wallis H test). Source data are provided as a Source Data file.

Supplemental Figure 3: Associations between immune mediator concentrations and COVID-19 severity risk in persons living with HIV (PLWH) and those without HIV.

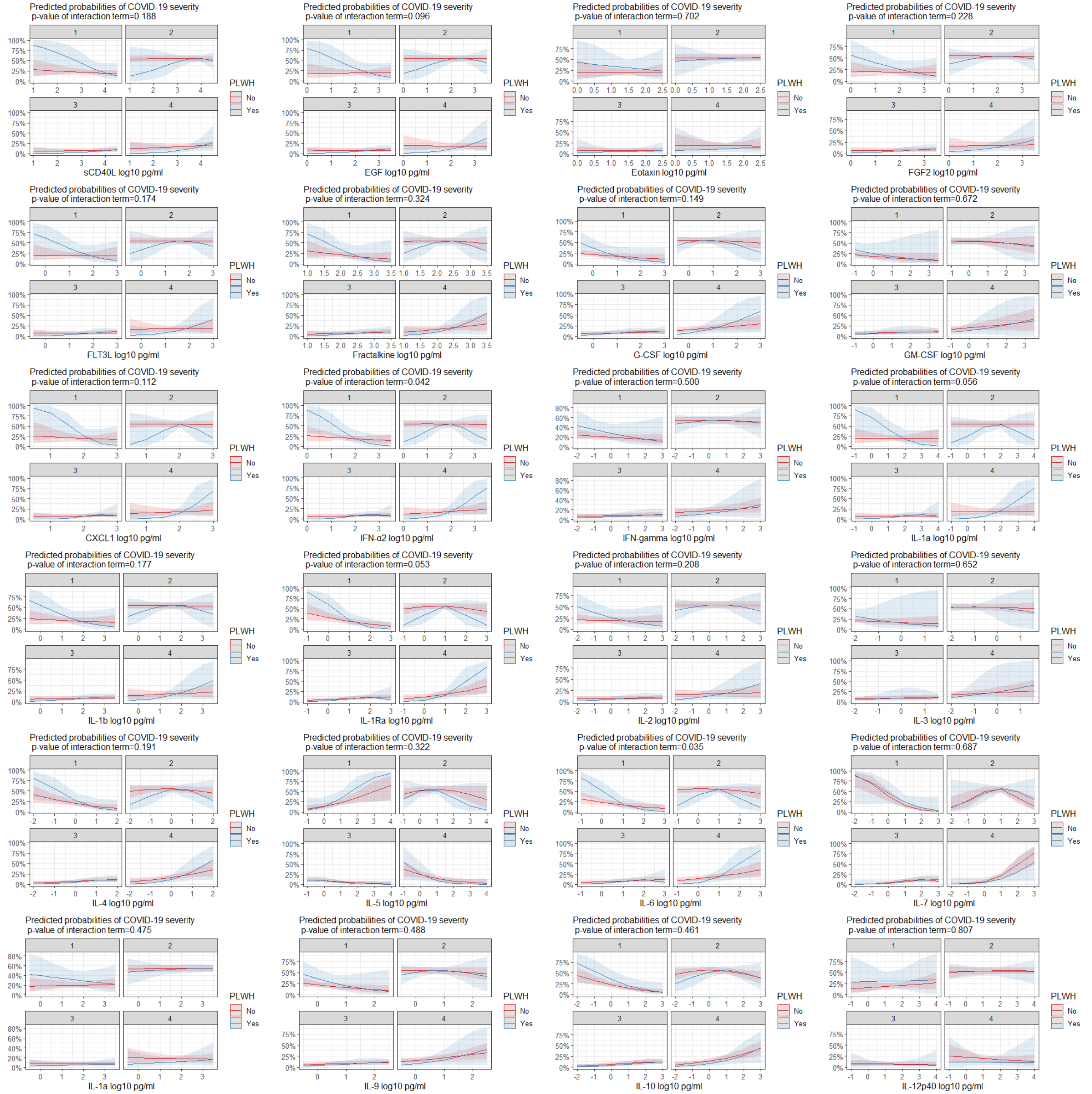

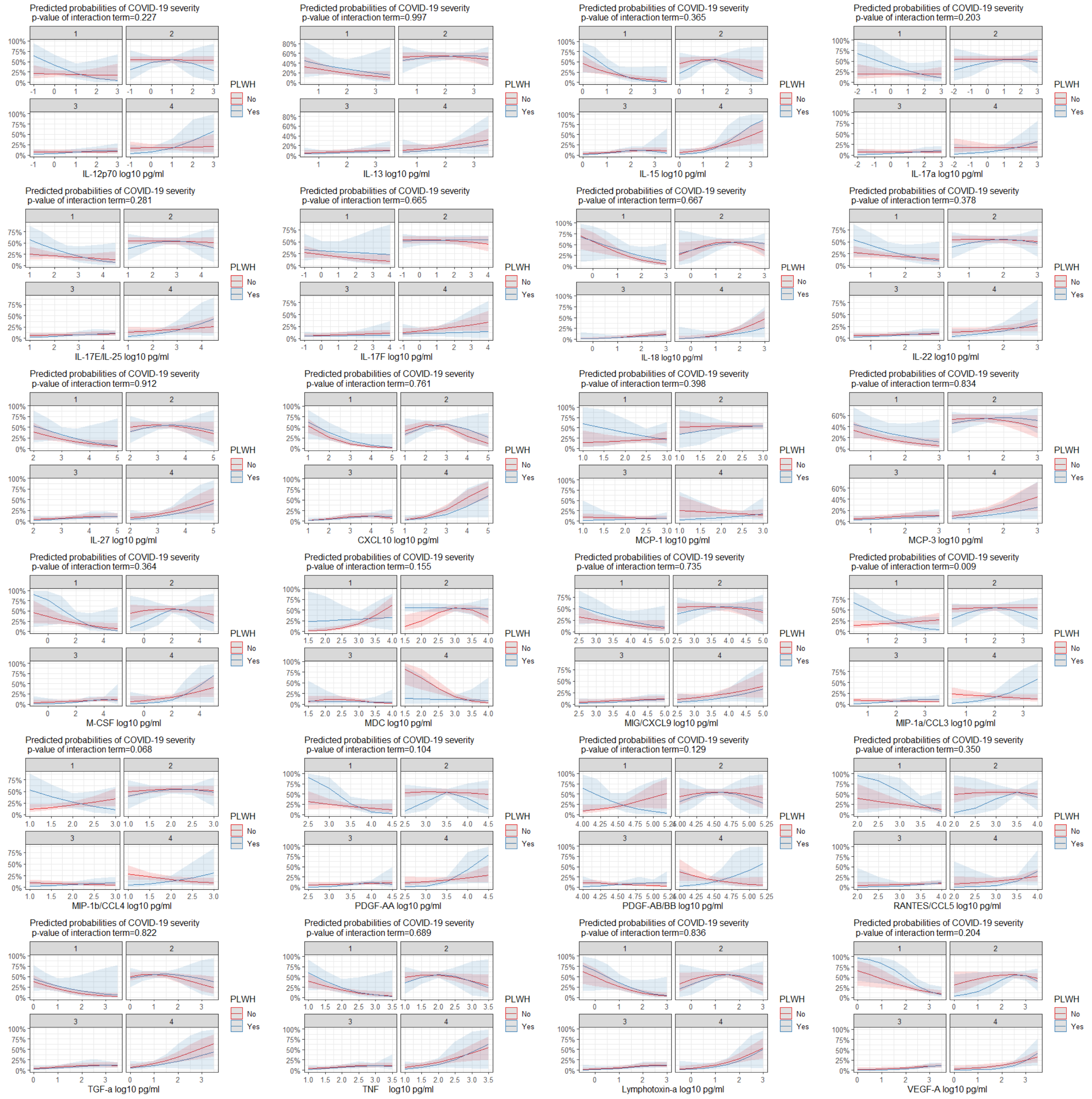

Each plot reflects multivariable proportional odds model including an interaction term for the product of log_{10} mediator concentration and HIV status, as well as age and sex; p-values reflect two-sided Wald test of interaction term (N=302; persons living with HIV, N=33; patients living without HIV, N=269; 4 patients without definitive assessment of HIV status excluded). 1=Asymptomatic, 2=Mild, 3=Moderate, 4=Severe. Source data are provided as a Source Data file.

Supplemental Figure 4: Inferred immune cell abundance as inferred from CIBERTSORTx digital cytometry deconvolution, stratified by COVID-19 severity (N=100).

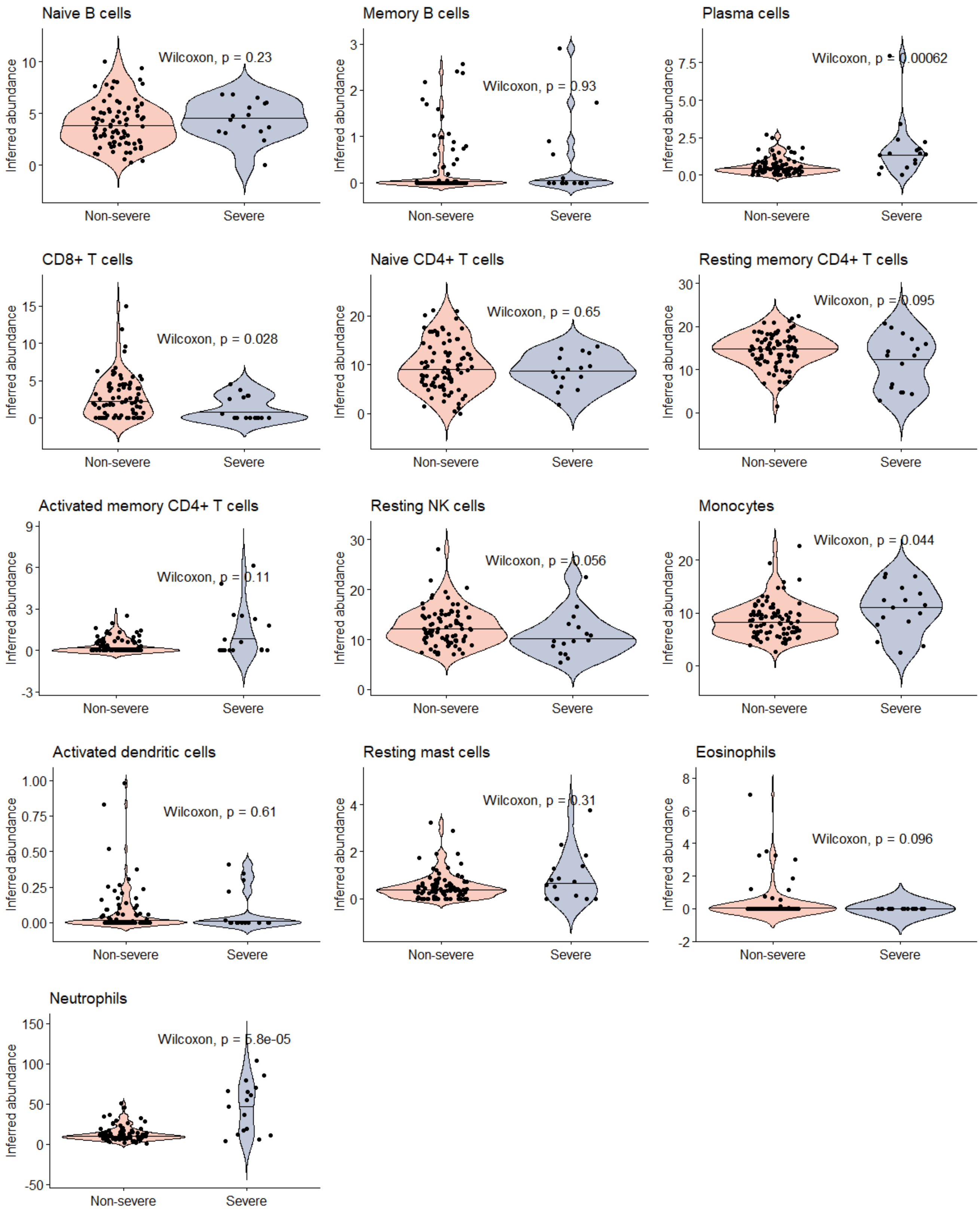

Source data are provided as a Source Data file.

Supplemental Figure 5: Strength and expected influence for each node in clinicomolecular force-directed correlation network among patients with symptomatic COVID-19 (N=240).

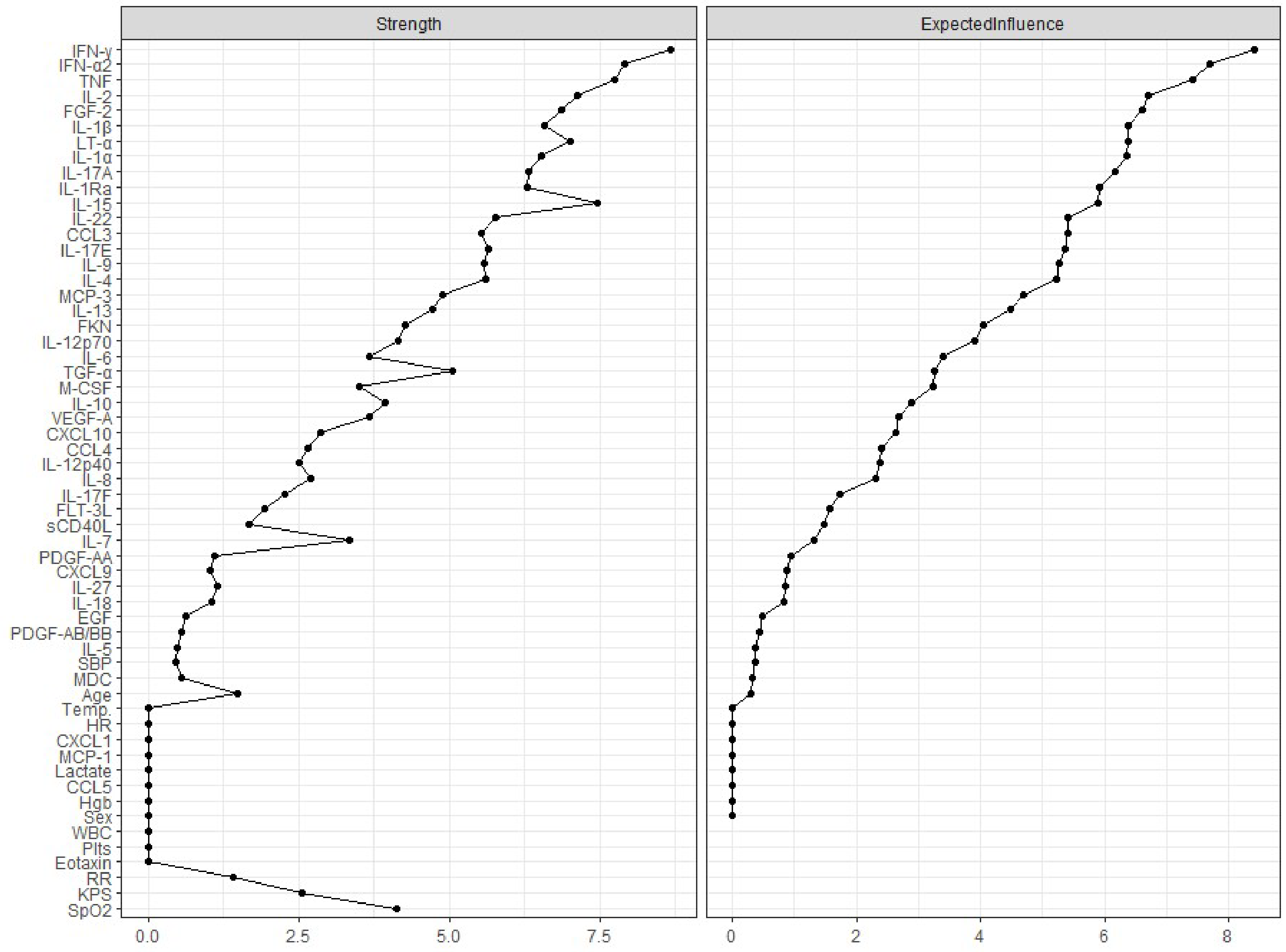

Source data are provided as a Source Data file.

Supplemental Figure 6: Hierarchical and force-directed network correlation plots including mediator and clinical data and HIV status among patients with symptomatic COVID-19.

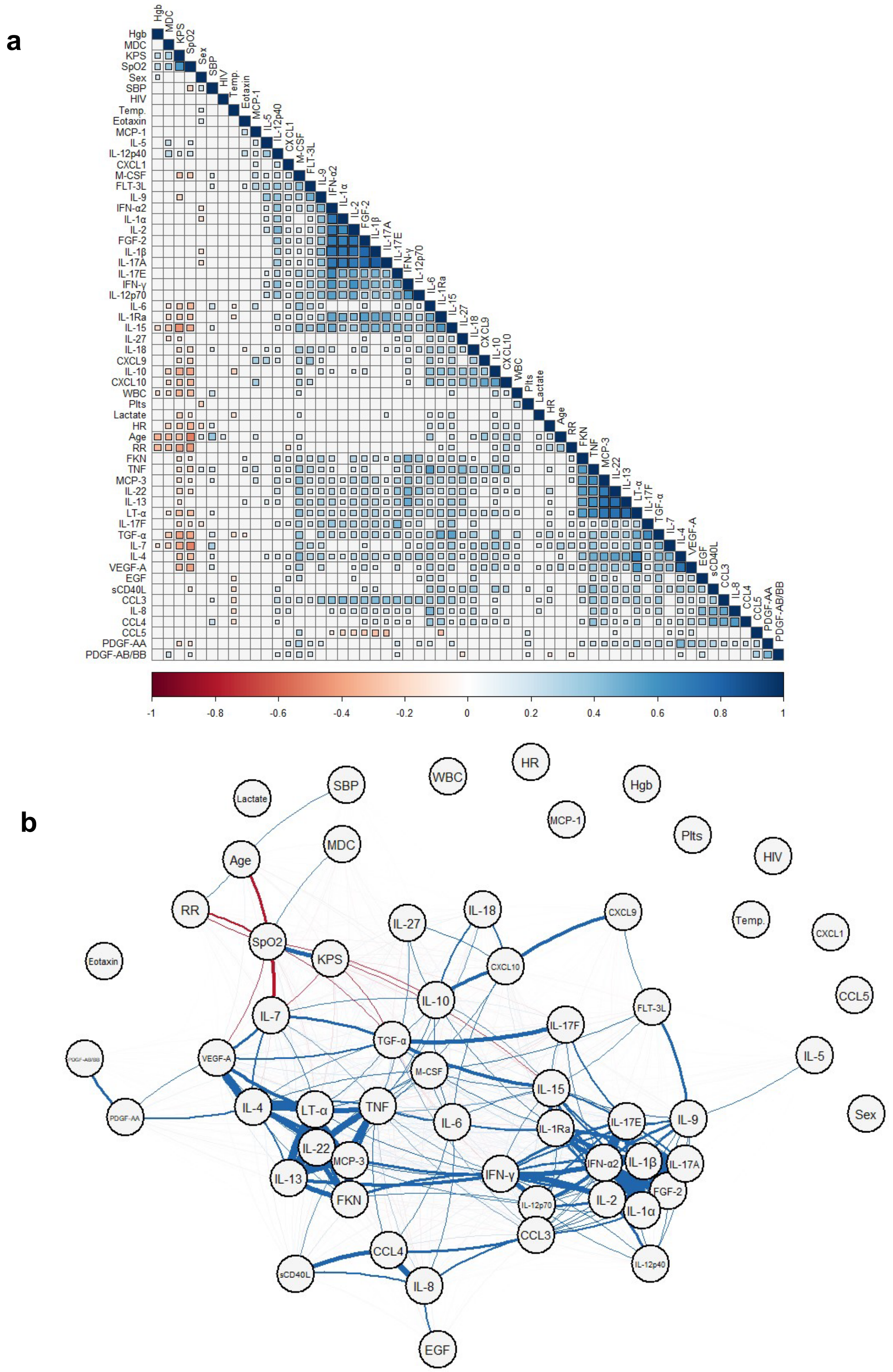

(a) Hierarchical correlation matrices showing relationships between demographics, clinical variables, HIV status, and immune mediators in patients with symptomatic COVID-19 (N=236; 4 patients without definitive HIV status assessment excluded); shaded squares reflect Spearman correlation coefficients filtered by a Benjamini-Hochberg adjusted p-value ≤0.05. **(b)** Force-directed weighted correlation network showing relationships between immune mediators, clinical variables, and HIV status in patients with symptomatic COVID-19 (N=236; 4 patients without definitive HIV status assessment excluded); network structured on weighted correlations with each variable set as a network node and between-variable correlations significant at a Benjamini-Hochberg-adjusted p-value ≤0.05 indicated by weighted edges (blue edges indicate positive correlation, red edges indicate negative correlation, edge width indicates strength of correlation based on Spearman correlation coefficient). Source data are provided as a Source Data file.

Supplemental Figure 7: Strength and expected influence for each node in clinicomolecular force-directed correlation network (with HIV status included as node) among patients with symptomatic COVID-19 (N=236; 4 patients without definitive HIV status assessment excluded).

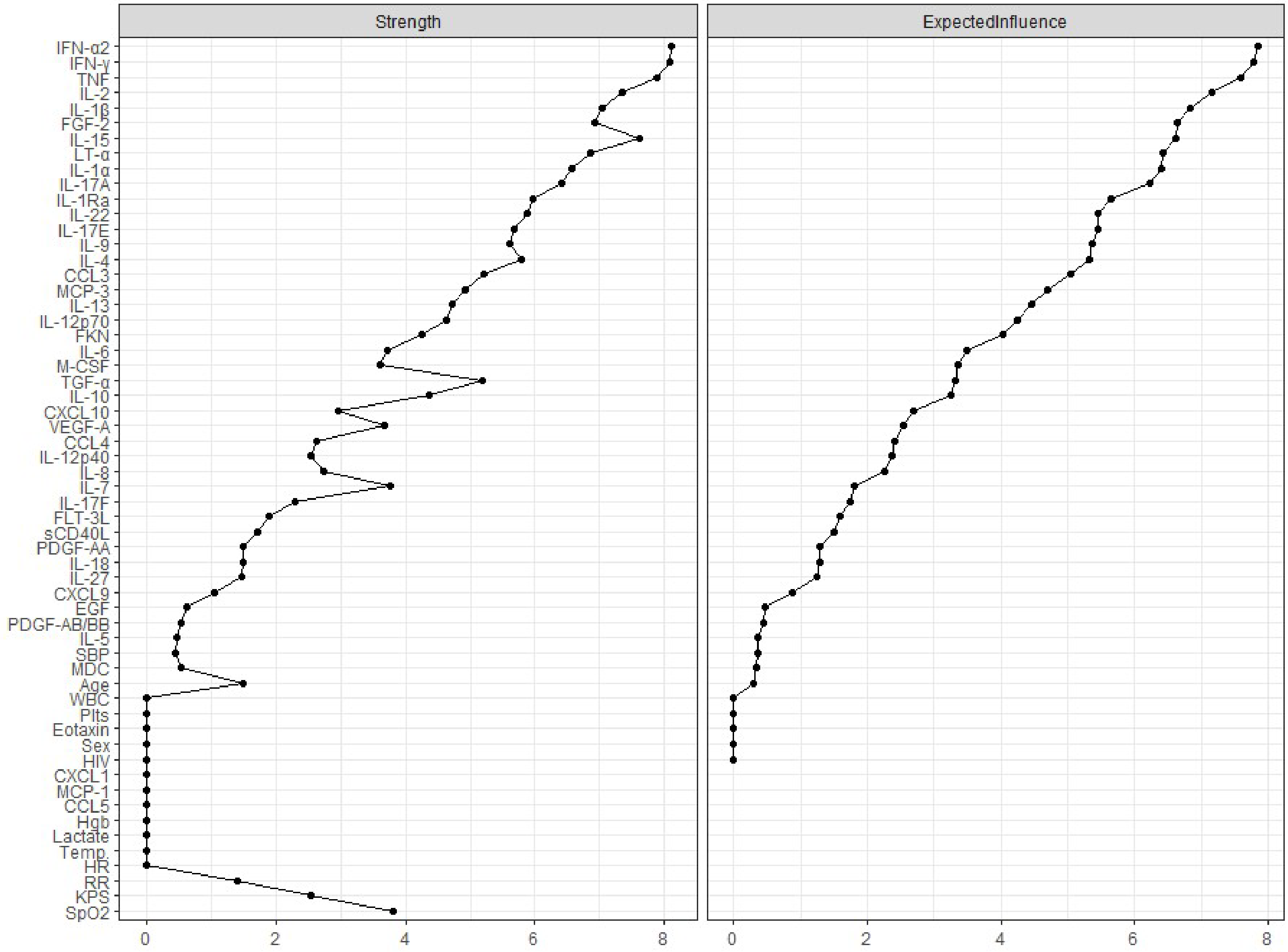

Source data are provided as a Source Data file.

Supplemental Figure 8: Unsupervised consensus k-means clustering of immune mediators in patients with symptomatic COVID-19 (N=240).

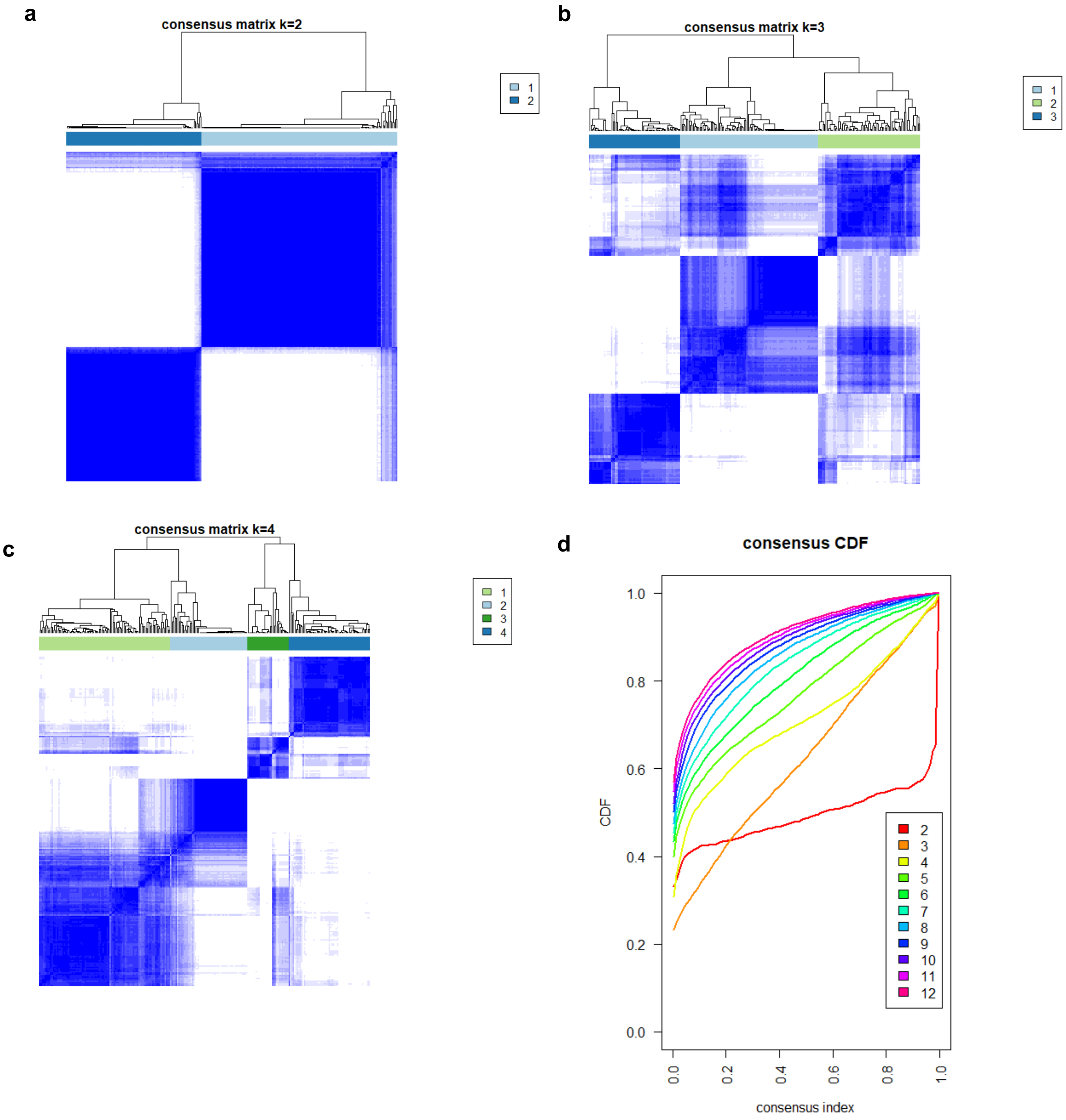

e

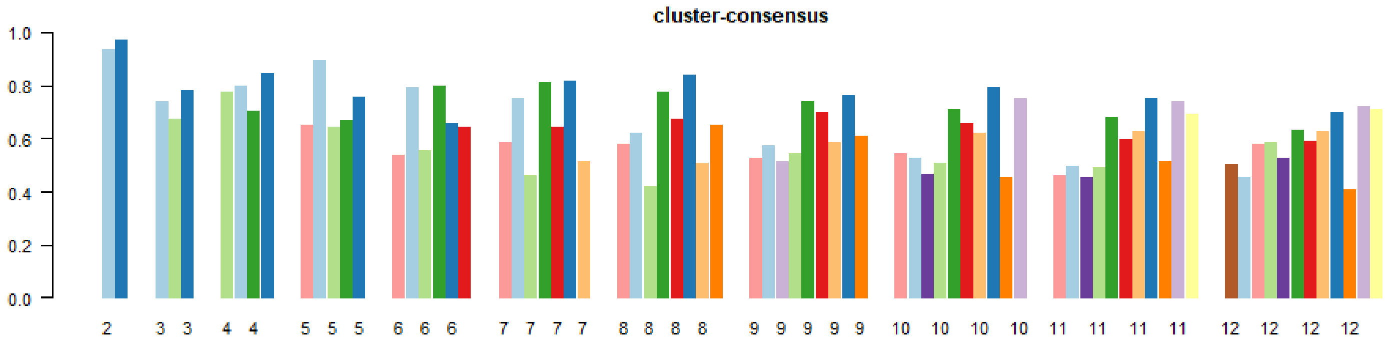

(a-c) Consensus matrices for cluster partitions of *k*=2 (optimal) and other representative examples *(k*=3 and *k*=4). **(d)** Consensus cumulative distribution function (CDF) plot, in which highest and more horizontal line reflects optimally stable partition. **(e)** Cluster consensus plot showing the average pairwise item consensus values between cluster members, with higher values across all bars suggestive of optimal *k.* Source data are provided as a Source Data file.
