# Supplementary material for: COVID-19 immune signatures in Uganda persist in HIV co-infection and diverge by pandemic phase
Source: Nat Commun. 2024 Feb 17;15:1475. doi: 10.1038/s41467-024-45204-3 (PMC10874401; doi:10.1038/s41467-024-45204-3)
Supplement: Supplementary file 3 — Description of Additional Supplementary Files [file 41467_2024_45204_MOESM3_ESM.pdf]

## Description of Additional Supplementary Files

**File: Supplementary Data File 1**

**Description**: Patient characteristics stratified by COVID-19 severity

**File: Supplementary Data File 2**

**Description:** Patient characteristics in RNAseq subcohort

**File: Supplementary Data File 3**

**Description:** Patient characteristics stratified by SARS-CoV-2-variant driven pandemic phase at time of hospitalization

**File: Supplementary Data File 4**

**Description:** Patient characteristics stratified by cluster-derived COVID-19 Response Signatures

**File: Supplementary Data File 5**

**Description:** Patient characteristics in influenza and non-influenza severe acute respiratory infection (SARI) cohort
