# Supplementary material for: COVID-19 immune signatures in Uganda persist in HIV co-infection and diverge by pandemic phase
Source: Nat Commun. 2024 Feb 17;15:1475. doi: 10.1038/s41467-024-45204-3 (PMC10874401; doi:10.1038/s41467-024-45204-3)
Supplement: Supplementary file 9 — Reporting Summary [file 41467_2024_45204_MOESM9_ESM.pdf]

nature portfolio

Corresponding author(s): Matthew Cummings

Last updated by author(s): Dec 21, 2023

Reporting Summary

Nature Portfolio wishes to improve the reproducibility of the work that we publish. This form provides structure for consistency and transparency in reporting. For further information on Nature Portfolio policies, see our Editorial Policies and the Editorial Policy Checklist.

Statistics

For all statistical analyses, confirm that the following items are present in the figure legend, table legend, main text, or Methods section.

n/a	Confirmed	
☐	☒	The exact sample size (*n*) for each experimental group/condition, given as a discrete number and unit of measurement
☐	☒	A statement on whether measurements were taken from distinct samples or whether the same sample was measured repeatedly
☐	☒	The statistical test(s) used AND whether they are one- or two-sided<br>*Only common tests should be described solely by name; describe more complex techniques in the Methods section.*
☐	☒	A description of all covariates tested
☐	☒	A description of any assumptions or corrections, such as tests of normality and adjustment for multiple comparisons
☐	☒	A full description of the statistical parameters including central tendency (e.g. means) or other basic estimates (e.g. regression coefficient) AND variation (e.g. standard deviation) or associated estimates of uncertainty (e.g. confidence intervals)
☐	☒	For null hypothesis testing, the test statistic (e.g. *F*, *t*, *r*) with confidence intervals, effect sizes, degrees of freedom and *P* value noted<br>*Give P values as exact values whenever suitable.*
☒	☐	For Bayesian analysis, information on the choice of priors and Markov chain Monte Carlo settings
☐	☒	For hierarchical and complex designs, identification of the appropriate level for tests and full reporting of outcomes
☐	☒	Estimates of effect sizes (e.g. Cohen's *d*, Pearson's *r*), indicating how they were calculated

Our web collection on statistics for biologists contains articles on many of the points above.

Software and code

Policy information about availability of computer code

Data collection	No software was used for data collection.
Data analysis	Analyses were performed using R (v3.6.1, R Foundation for Statistical Computing, Vienna, Austria) including the following packages: ComplexHeatmap, ggpubr, rstatix, ordinal, sjPlot, jtools, xgboost, caret, corrplot, qgraph, fdrtool, ConsensusClusterPlus, NbClust, FactoMineR, factoextra, PLSDAbatch, ComplexUpset, tidycmprsk, ggsurvfit, DESeq2, and mice. Biological pathway analysis of RNAseq data was performed using the Gene Set Enrichment Analysis platform (v4.2).

For manuscripts utilizing custom algorithms or software that are central to the research but not yet described in published literature, software must be made available to editors and reviewers. We strongly encourage code deposition in a community repository (e.g. GitHub). See the Nature Portfolio guidelines for submitting code & software for further information.

Data

Policy information about availability of data

All manuscripts must include a data availability statement. This statement should provide the following information, where applicable:

- Accession codes, unique identifiers, or web links for publicly available datasets
- A description of any restrictions on data availability
- For clinical datasets or third party data, please ensure that the statement adheres to our policy

Raw bead-based proteomics data have been deposited in Dryad under accession code DOI: 10.5061/dryad.2bvq83bvd (https://datadryad.org/stash/dataset/

doi:10.5061/dryad.2bvq83bvd). Raw RNAseq data have been deposited in NIH/NCBI Sequence Read Archive and dbGaP under accession numbers PRJNA950542 (https://www.ncbi.nlm.nih.gov/bioproject?LinkName =sra_bioproject&from_uid=27274595) and phs003246.v1.p1 (https://www.ncbi.nlm.nih.gov /projects/gap/cgi-bin/study.cgi?study_id=phs003246.v1.p1), respectively. In concordance with participant consent and institutional certification of genomic data sharing, raw RNAseq data are available to investigators with an IRB approved protocol. Requests for access can be made to the NIH/NIAID Data Access Committee (niaid_datasharing@niaid.nih.gov). Source data are provided with this paper.

Research involving human participants, their data, or biological material

Policy information about studies with human participants or human data. See also policy information about sex, gender (identity/presentation), and sexual orientation and race, ethnicity and racism.

Reporting on sex and gender	Our prospective cohort study enrolled consecutive patients admitted to a national COVID-19 referral hospital in Uganda with laboratory-confirmed SARS-CoV-2 infection. Both males and females were enrolled; there was no preference for enrollment by self-reported sex. 76% (231/306) of enrolled COVID-19 patients were male. Disaggregated data including self-reported sex is provided with both the raw dataset and our Source Data file. Where indicated, multivariable models were adjusted for self-reported sex (among other variables).
Reporting on race, ethnicity, or other socially relevant groupings	All patients enrolled in our study were Ugandan and self-identified as black and non-Hispanic/Latino.
Population characteristics	The median age of enrolled participants was 37 years (interquartile range 28, 46) and 76% (231/306) were male. 11% (33/302) were living with HIV, 14% (42/306) had hypertension, and 3% (10/306) had diabetes. Based on World Health Organization (WHO) criteria, we stratified patients into four groups of clinical severity: asymptomatic (N=66 [21.6%]), mild (N=149 [48.7%]), moderate (N=21 [6.9%]), and severe (N=70 [22.9%]). Among patients with severe COVID-19, 90.0% (63/70) received oxygen therapy and 88.6% (62/70) received corticosteroids.
Recruitment	Patients were prospectively enrolled in this study if they were: (1) admitted to Entebbe Regional Referral Hospital during the study period, (2) 5 years of age, (3) had laboratory-confirmed SARS-CoV-2 infection by polymerase-chain-reaction (PCR) testing of a naso-/oropharyngeal swab sample at a Ministry of Health-accredited laboratory, and (4) were able to provide written informed consent or had a surrogate available to do so. Pregnant females were excluded. During each pandemic wave in Uganda (differentiated by varying levels of community transmission and epidemic peaks), admissions to our study site (a national COVID-19 referral hospital) were screened for eligibility by study staff on a daily basis. Although patients 5-17 years of age were enrolled, given variations in normal physiologic parameters, COVID-19 severity criteria, and SARS-CoV-2 host responses across age groups, for analyses reported in this manuscript we included only adults (age ≥18 years).
Ethics oversight	Each enrolled participant or their surrogate provided written informed consent. Study protocols were approved by ethics committees at Columbia University (AAAR1450), Uganda Virus Research Institute (GC/127/17/02-06/582), and Uganda National Council for Science and Technology (HS2308).

Note that full information on the approval of the study protocol must also be provided in the manuscript.

Field-specific reporting

Please select the one below that is the best fit for your research. If you are not sure, read the appropriate sections before making your selection.

☒ Life sciences ☐ Behavioural & social sciences ☐ Ecological, evolutionary & environmental sciences

For a reference copy of the document with all sections, see nature.com/documents/nr-reporting-summary-flat.pdf

Life sciences study design

All studies must disclose on these points even when the disclosure is negative.

Sample size	The sample size of our study was determined based on the time period of enrollment (March 22nd, 2020 to July 14th, 2021), which corresponded to the emergence of SARS-CoV-2 in Uganda and the occurrence of multiple pandemic waves in the country.
Data exclusions	Although patients 5-17 years of age were enrolled in the parent cohort, given variations in normal physiologic parameters, COVID-19 severity criteria, and SARS-CoV-2 host responses across age groups, for the analyses reported here we included only adults (age 18 years).
Replication	For multiplexed immunoassays, soluble mediators were quantified in each sample in duplicate with the mean concentration used for analysis. Replications were successful in 94% of attempts. For unsuccessful replications, the single generated value was used for analysis. A single RNAseq run was performed for each sample.
Randomization	For multiplexed immunoassays, samples from different pathogen and severity groups were randomized across plates. For RNAseq of COVID-19 patients, samples were randomized by severity group prior to RNA extraction, library preparation, and sequencing.
Blinding	For multiplexed immunoassays, samples were analyzed by technicians blinded to pathogen and severity status. For RNAseq, sequencing was performed by technicians blinded to severity status. At the time of blood sample collection, study staff were not blinded to pathogen and severity status as patients were known to have COVID-19 and their symptomatic status, vital signs, and levels of respiratory support were apparent at the bedside.

Reporting for specific materials, systems and methods

We require information from authors about some types of materials, experimental systems and methods used in many studies. Here, indicate whether each material, system or method listed is relevant to your study. If you are not sure if a list item applies to your research, read the appropriate section before selecting a response.

Materials & experimental systems

n/a	Involved in the study
☒	☐ Antibodies
☒	☐ Eukaryotic cell lines
☒	☐ Palaeontology and archaeology
☒	☐ Animals and other organisms
☐	☒ Clinical data
☒	☐ Dual use research of concern
☒	☐ Plants

Methods

n/a	Involved in the study
☒	☐ ChIP-seq
☐	☐ Flow cytometry
☒	☐ MRI-based neuroimaging

Clinical data

Policy information about clinical studies

All manuscripts should comply with the ICMJE guidelines for publication of clinical research and a completed CONSORT checklist must be included with all submissions.

Clinical trial registration

N/A

Study protocol

Data and sample collection protocols for our observational study were based on the Tier 1 approach of the COVID-19 Clinical Characterization Protocol (CCP) developed by the International Severe Acute Respiratory and Emerging Infection Consortium (ISARIC) and WHO. The protocol is linked at https://isaric.net/ccp/.

Data collection

We enrolled patients with laboratory (PCR)-confirmed SARS-CoV-2 infection admitted to Entebbe Regional Referral Hospital (ERRH), a public hospital in central Uganda. During the study period, ERRH functioned as a national referral hospital for COVID-19; patients with SARS-CoV-2 infection nationwide were referred to the facility for management. Patients were enrolled from March 22nd, 2020 to July 14th, 2021.

Outcomes

The primary clinical outcome of our study was the frequency of severe COVID-19, defined as the proportion of all enrolled SARS-CoV-2 admissions who fulfilled criteria for severe COVID-19. Based on WHO guidelines, we considered adult patients to have severe COVID-19 if they met 1 of the following criteria: (1) oxygen saturation 90%, (2) respiratory rate 30 breaths/minute, (3) showed signs of respiratory distress [chest indrawing, nasal flaring, grunting respirations], (4) received oxygen-therapy. The secondary study outcome was a composite measure of in-hospital outcome (death in-hospital or transfer to Uganda's highest level referral hospital due to progressive illness severity).

Plants

Seed stocks

N/A

Novel plant genotypes

N/A

Authentication

N/A

Flow Cytometry

Plots

Confirm that:

- [ ] The axis labels state the marker and fluorochrome used (e.g. CD4-FITC).
- [ ] The axis scales are clearly visible. Include numbers along axes only for bottom left plot of group (a 'group' is an analysis of identical markers).
- [ ] All plots are contour plots with outliers or pseudocolor plots.
- [ ] A numerical value for number of cells or percentage (with statistics) is provided.

Methodology

Sample preparation	*Describe the sample preparation, detailing the biological source of the cells and any tissue processing steps used.*
Instrument	*Identify the instrument used for data collection, specifying make and model number.*
Software	*Describe the software used to collect and analyze the flow cytometry data. For custom code that has been deposited into a community repository, provide accession details.*
Cell population abundance	*Describe the abundance of the relevant cell populations within post-sort fractions, providing details on the purity of the samples and how it was determined.*
Gating strategy	*Describe the gating strategy used for all relevant experiments, specifying the preliminary FSC/SSC gates of the starting cell population, indicating where boundaries between "positive" and "negative" staining cell populations are defined.*

- [ ] Tick this box to confirm that a figure exemplifying the gating strategy is provided in the Supplementary Information.
